# The Mesencephalic Locomotor Region: Multiple Cell Types, Multiple Behavioral Roles, and Multiple Implications for Disease

**DOI:** 10.1177/10738584221139136

**Published:** 2022-12-28

**Authors:** Dimitri Ryczko

**Affiliations:** 1 Département de Pharmacologie-Physiologie, Faculté de médecine et des sciences de la santé, Université de Sherbrooke, Sherbrooke, Canada; 2 Centre de recherche du Centre Hospitalier Universitaire de Sherbrooke, Sherbrooke, Canada; 3 Neurosciences Sherbrooke, Sherbrooke, Canada; 4 Institut de Pharmacologie de Sherbrooke, Sherbrooke, Canada

**Keywords:** locomotion, gait freezing, mesencephalic locomotor region, deep brain stimulation, cuneiform nucleus, pedunculopontine nucleus, Parkinson disease

## Abstract

The mesencephalic locomotor region (MLR) controls locomotion in vertebrates. In humans with Parkinson disease, locomotor deficits are increasingly associated with decreased activity in the MLR. This brainstem region, commonly considered to include the cuneiform and pedunculopontine nuclei, has been explored as a target for deep brain stimulation to improve locomotor function, but the results are variable, from modest to promising. However, the MLR is a heterogeneous structure, and identification of the best cell type to target is only beginning. Here, I review the studies that uncovered the role of genetically defined MLR cell types, and I highlight the cells whose activation improves locomotor function in animal models of Parkinson disease. The promising cell types to activate comprise some glutamatergic neurons in the cuneiform and caudal pedunculopontine nuclei, as well as some cholinergic neurons of the pedunculopontine nucleus. Activation of MLR GABAergic neurons should be avoided, since they stop locomotion or evoke bouts flanked with numerous stops. MLR is also considered a potential target in spinal cord injury, supranuclear palsy, primary progressive freezing of gait, or stroke. Better targeting of the MLR cell types should be achieved through optimized deep brain stimulation protocols, pharmacotherapy, or the development of optogenetics for human use.

In vertebrates, a key brain region involved in the control of locomotion is the mesencephalic locomotor region (MLR; Shik and others 1966). Located at the border between the mesencephalon and rhombencephalon, it controls locomotor initiation, locomotor speed, gait transitions, and even locomotor arrests (Arber and Costa 2022; for recent review, Ferreira-Pinto and others 2018; Grillner and El Manira 2020; Leiras and others 2022; Noga and Whelan 2022). The MLR is strongly conserved in vertebrates (Figure 1A–E). Electrical stimulation of the MLR controls locomotor output in lamprey (Sirota and others 2000; Figure 1F), zebrafish (Carbo-Tano and others 2022; Figure 1G), stingray (Bernau and others 1991), salamander (Cabelguen and others 2003; Figure 1H), rat (Bachmann and others 2013; Garcia-Rill and others 1987; Hofer and others 2022; Figure 1I), mouse (Roseberry and others 2016), rabbit (Musienko and others 2008), goose (Steeves and others 1987), pig (Chang, Santamaria, and others 2021), cat (Opris and others 2019; Shik and others 1966), and monkey (Eidelberg and others 1981; Gay and others 2020; Goetz and others 2016). It does so by providing direct glutamatergic input to reticulospinal neurons in the reticular formation—as demonstrated in lamprey (Brocard and Dubuc 2003; Le Ray and others 2003), zebrafish (Carbo-Tano and others 2022), salamander (Ryczko, Auclair, and others 2016), and mouse (Bretzner and Brownstone 2013; Capelli and others 2017)—which send direct input to interneurons of the central pattern generator for locomotion, as shown in lamprey (Buchanan and Grillner 1987; for review, Grillner and El Manira 2020; Leiras and others 2022; Figure 1A). In humans, the MLR is activated when people are asked to imagine that they are walking (Karachi and others 2010), and locomotor deficits appear if the MLR is damaged (Demain and others 2014; Kuo and others 2008; Masdeu and others 1994).

**Figure 1.**
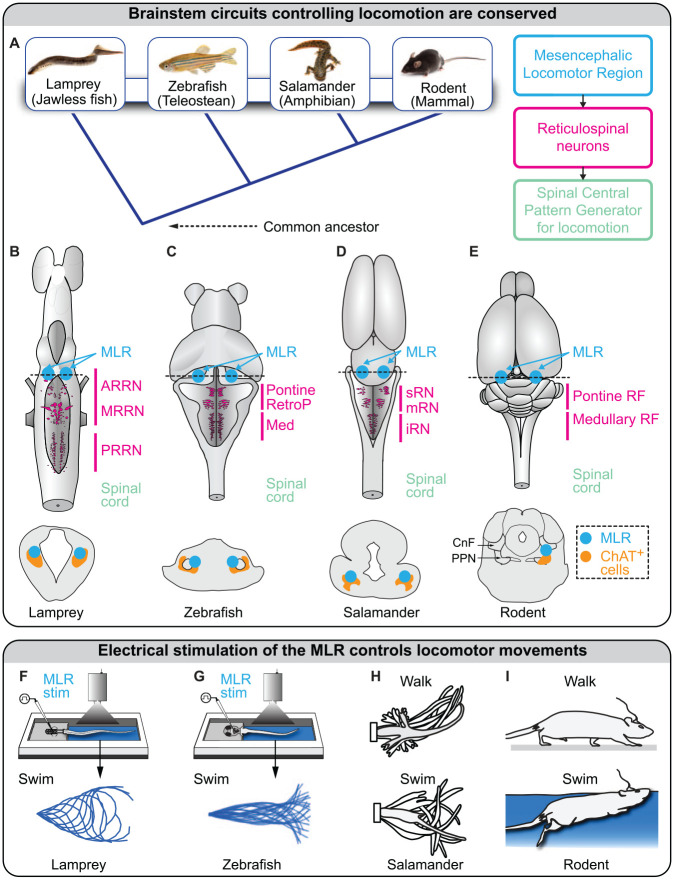
Brainstem locomotor circuits are evolutionary conserved. (A) Left: vertebrate evolution from lamprey to mammals. Right: the three basic layers of the locomotor circuitry conserved in vertebrates. (B–E) Brains of lamprey (adapted from Ryczko and others 2020), zebrafish (adapted from Carbo-Tano and others 2022), salamander (Ryczko, Auclair, and others 2016), and rodent (adapted from Ryczko, Cone, and others 2016). In mammals, many reticulospinal neurons are distributed in the pontine and medullary reticular formations (RFs). Possible homologs of the mammalian pontine RFs are highlighted in lamprey (anterior rhombencephalic reticular nucleus [ARRN], middle rhombencephalic reticular nucleus [MRRN]), zebrafish (pontine reticular formation, retropontine reticular formation [RetroP]), and salamander (superior reticular nucleus [sRN], middle reticular nucleus [mRN]). Possible homologs of the mammalian medullary RFs are also highlighted in lamprey (posterior rhombencephalic reticular nucleus [PRRN]), zebrafish (medullary reticular formation), and salamander (inferior reticular nucleus [iRN]). Bottom: schematic illustrations of transverse slices at the level of the mesencephalic locomotor region (MLR). Efficient MLR stimulation sites colocalize with cholinergic neurons (positive for choline acetyltransferase [ChAT]) of the pedunculopontine nucleus in mammals, the pedunculopontine nucleus and laterodorsal tegmental nucleus in lamprey and salamander, and a putative homolog ChAT^+^ cell group in zebrafish. This indicates that MLR neurons generating the locomotor drive are consistently located around the cholinergic population in vertebrates. (F–H) Electrical stimulation of the MLR evokes swimming in a lamprey semi-intact preparation, in which the brain is accessible and the body is free to move (adapted from Brocard and others 2010; Ryczko and others 2020) and in larval zebrafish with the head embedded in agarose and the body free to move (adapted from Carbo-Tano and others 2022). In a salamander semi-intact preparation, low-intensity MLR stimulation evokes walking, and higher stimulation intensities evoke swimming (adapted from Cabelguen and others 2003). In rats and mice, electrical stimulation of the MLR evokes walking movements on ground and swimming movements underwater (adapted from Bachmann and others 2013; see also Roussel and others 2022). CnF, cuneiform nucleus; PPN, pedunculopontine nucleus.

Because of its ubiquity in vertebrates, the MLR became a potential target to stimulate in diseases with locomotor deficits. For instance, in Parkinson disease (PD), locomotor deficits (akinesia, gait freezing) are associated with decreased activity in the MLR (for review, Ryczko and Dubuc 2017; Shine and others 2013; Thevathasan and others 2012), suggesting that an increase in MLR activity could alleviate locomotor deficits. Since 2005, the MLR has been explored as a target for deep brain stimulation (DBS) to improve locomotor function in PD (Mazzone and others 2005; Plaha and Gill 2005). The outcome of the initial studies was positive, but these results were not systematically reproduced (e.g., Garcia-Rill and others 2019; Thevathasan and others 2018).

A possible explanation to such variability is that the MLR is a heterogeneous structure. In mammals, it mainly contains the cuneiform nucleus (CnF) dorsally and the pedunculopontine nucleus (PPN) ventrally (Figure 2). Most clinical studies targeted the PPN, leaving the CnF largely unexplored (Chang and others 2020; Goetz and others 2019). These nuclei contain different genetically defined cell types with opposing effects on locomotion. The CnF contains mainly glutamatergic neurons (positive for the vesicular glutamatergic transporter 2 [Vglut2]) and GABAergic neurons (positive for the vesicular GABA transporter [VGAT]). The PPN contains glutamatergic, GABAergic, and cholinergic neurons (positive for choline acetyl transferase [ChAT], an enzyme that synthetizes acetylcholine; Roseberry and others 2016; Figure 2). Although not reviewed here, MLR cells also coexpress neuropeptides, and this likely increases the heterogeneity of cell types (for review, Ryczko and Dubuc 2013). DBS protocols rely on electrical stimulation, which is not yet optimized to selectively activate the MLR neurons that generate the locomotor drive. During the last 8 years, studies in rodents, mainly based on optogenetics and chemogenetics (Box 1), allowed researchers to start dissecting the role of each genetically defined cell type, and the results indicate that some MLR neurons initiate locomotion while others stop locomotion (Figure 2). DBS likely activates a heterogeneous population of neurons around the electrode, including the ones that stop locomotion, and this likely contributes to the variable outcomes. Hence, cell type–specific targeting should refine the behavioral outcomes evoked by MLR stimulation.

**Figure 2.**
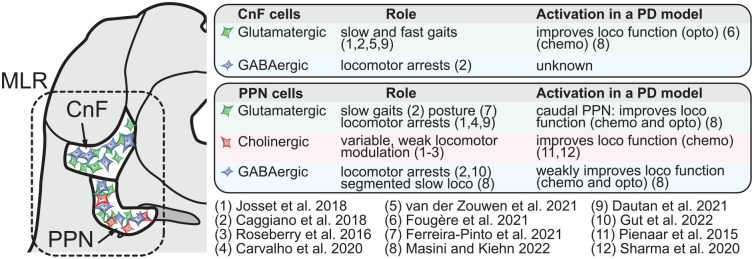
Effects of mesencephalic locomotor region (MLR) cell type stimulation in intact rodents and in rodent models of Parkinson disease (PD). Left: illustration of a slice of the mouse brain at the level of the MLR. The dorsal part (cuneiform nucleus [CnF]) and the ventral part (pedunculopontine nucleus [PPN]) are delineated. On the right, the locomotor (loco) effects resulting from optogenetic (opto) or chemogenetic (chemo) activation of MLR glutamatergic neurons (positive for vesicular glutamate transporter 2 [Vglut2]), GABAergic neurons (positive for vesicular GABA transporter [VGAT]), or cholinergic neurons (positive for choline acetyltransferase [ChAT]) in intact rodents and in rodent animal models of PD. Note that some authors targeted MLR CamKIIa^+^ neurons (e.g., Carvalho and others 2020), among which many but not all are glutamatergic (Kreeger and others 2021; Meng and others 2016; Roseberry and others 2016; Wang and others 2013). Also note that the first studies targeted the MLR at large, without precisely targeting the CnF or PPN (Capelli and others 2017; Lee and others 2014; Roseberry and others 2016).

Box 1. **Selectivity and time scales of Electrical Stimulation versus Optogenetic or Chemogenetic Stimulation.**

(A) When one aims to stimulate a specific cell population, electrical stimulation has limited selectivity because the current delivered spreads all around the electrode, thus recruiting neurons nearby the electrode. The effects of electrical deep brain stimulation (DBS) are in the order of the millisecond, as shown by the spiking activity illustrated at the bottom.

(B) With optogenetics, genetically defined cell populations are made to express a light-sensitive ion channel, which makes those cells sensitive to light. Then, when light is shone in the target region, only the neurons expressing the light-sensitive ion channel are activated. Some light-sensitive ion channels can be used to activate neurons—such as channelrhodopsin (ChR2), sensitive to ~470-nm blue light, or ChrimsonR, a red-shifted channelrhodopsin sensitive to ~590-nm yellow light, whereas other ones can be used to inhibit neurons, including halorhodopsin, a chloride pump sensitive to ~580-nm yellow light (for review, Kim and others 2017). In terms of time scale, optogenetics is precise and allows the experimenter to switch neurons on or off from a few milliseconds to dozens of seconds or minutes.

(C) With chemogenetics, genetically defined cell populations are made to express a modified version of the human muscarinic receptor, which is sensitive to a drug (clozapine N-oxide) designed to activate this receptor. These receptors are thus called designer receptors exclusively activated by designer drugs (DREADD). Then, when the designer drug is delivered in the target brain region, only the cells expressing the DREADD will be activated. Some DREADD can be used to activate neurons, whereas others can be used to inhibit neurons (for review, Roth 2016). In terms of time scale, chemogenetics allows tonic activation or inhibition of the target neurons during dozens of minutes to an hour.

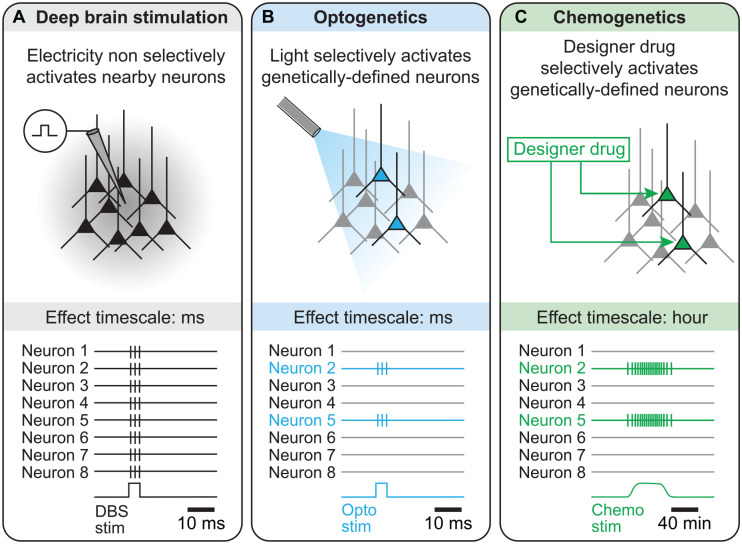

To help define the best cell type to stimulate in the MLR to improve locomotor function in pathologic conditions, here I review recent studies that reported the effects of selective stimulation of MLR cell types in intact animals and in animal models of PD. For further reading about the MLR, I refer to recent reviews on brainstem circuits controlling locomotor initiation, speed, stop, and steering movements (Leiras and others 2022), on the functional relations between brainstem motor circuits and the basal ganglia (Arber and Costa 2022), on MLR DBS in spinal cord injury (Noga and Guest 2021) and on state-dependent control of locomotion by brainstem motor circuits (Noga and Whelan 2022; Pernia-Andrade and others 2021).

## Targeting the MLR at Large by Using Molecular Tools: The First Studies

The first optogenetic stimulation of the MLR at large (i.e., without precisely targeting the CnF or PPN) was done by injecting in the MLR an adeno-associated virus driving the expression of channelrhodopsin (Box 1) under control of the calcium/calmodulin-dependent protein kinase IIa promoter (CamKIIa; Lee and others 2014). CamKIIa is expressed in glutamatergic neurons (Vglut2^+^) but also in a minority of nonglutamatergic ones (Kreeger and others 2021; Meng and others 2016; Roseberry and others 2016; Wang and others 2013). Optogenetic stimulation of MLR CamKIIa^+^ neurons evoked locomotion in head-fixed mice placed on a trackball (Lee and others 2014). In this study, the firing of a fraction of MLR cells was correlated with locomotor speed. Optogenetic activation of CamKIIa^+^ MLR cells also increased visually evoked responses in the visual cortex, though ascending projections to cholinergic neurons in the basal forebrain that send input to the visual cortex (Lee and others 2014).

Roseberry and others (2016) performed selective optogenetic activation of Vglut2^+^, VGAT^+^, or ChAT^+^ neurons of the MLR at large using transgenic mice coupled with adeno-associated virus injections. In head-fixed mice placed on a trackball, they showed the following: 1) optogenetic activation of MLR Vglut2^+^ neurons evoked locomotion and control locomotor speed; 2) the electrophysiologic activity of a fraction of Vglut2^+^ neurons correlated with locomotor speed; 3) optogenetic inhibition of MLR CamKIIa^+^ neurons decreased locomotor speed; 4) optogenetic activation of MLR VGAT^+^ neurons stopped locomotion; and 5) optogenetic activation of MLR ChAT^+^ neurons slightly increased the speed of ongoing locomotion. They also established that activation of striatal neurons of the direct and indirect pathways respectively increased or decreased locomotion by controlling the firing of CamKIIa^+^ MLR neurons.

Capelli and others (2017) identified some of the downward targets of MLR Vglut2^+^ neurons. Using adeno-associated virus injections in Vglut2-cre mice, they showed that ablation of Vglut2^+^ neurons in the lateral paragigantocellular nucleus (LPGi) reduced the speed of locomotion evoked by optogenetic stimulation of Vglut2^+^ MLR neurons in freely moving mice. Altogether these studies provided key information about the rodent MLR and laid the foundation for further dissection of the genetically defined cell populations in the PPN and CnF, as described below.

## CnF Vglut2^+^ Neurons: Slow and Fast Gaits

### Stimulation

**Figure 3.**
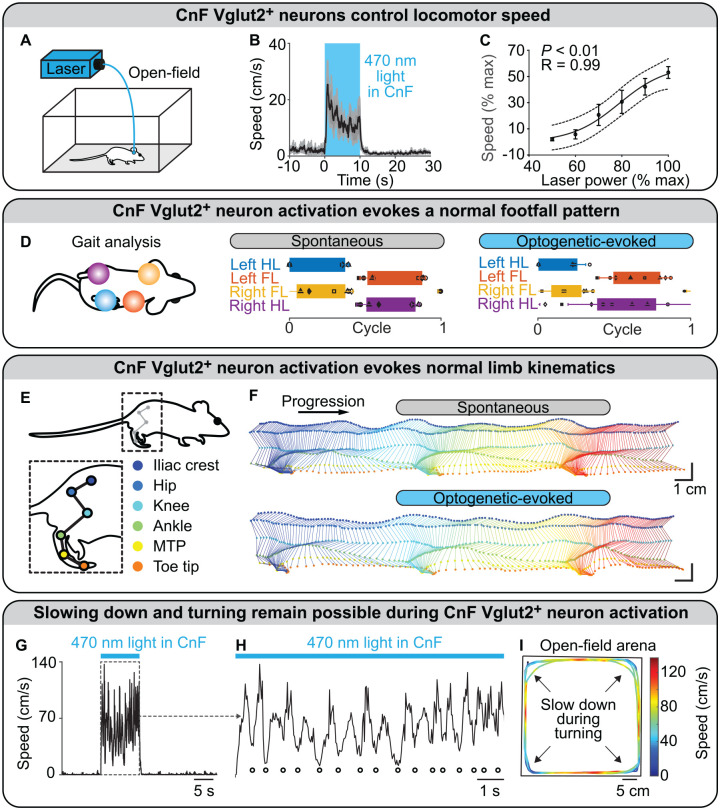
Optogenetic stimulation of glutamatergic neurons in the cuneiform nucleus controls locomotion in intact mice. (A–C) In an open field, blue light (470 nm) shone with a laser in the cuneiform nucleus (CnF) of mice expressing the ion-sensitive channel channelrhodopsin in glutamatergic neurons (positive for the vesicular glutamatergic transporter 2 [Vglut2]) evoked locomotion. Increasing laser power increased locomotor speed. Each data point is the average of 3 to 5 mice. (B) Data are presented as mean (black) ± SEM (grey). (C) Data are presented as mean ± SEM. Black line, sigmoidal fit; dotted lines, 95% prediction intervals. (D) In a linear corridor, the footfall pattern evoked by optogenetic stimulation of CnF Vglut2^+^ neurons was similar to that recorded during spontaneous locomotion. Each rectangle represents the normalized duration of the stance phase for each limb. (E, F) In a linear corridor, the hindlimb kinematics evoked by optogenetic stimulation of CnF Vglut2^+^ neurons were similar to those recorded during spontaneous locomotion. (F) Time elapsed from first to last frame is 700 ms (top) and 500 ms (bottom). (G, H) During tonic optogenetic stimulation of CnF Vglut2^+^ neurons, multiple slow-downs were smoothly achieved (highlighted with white filled dots). (I) The slow-downs were happening when the mouse was approaching the walls of the arena, indicating that dynamic integration of visual inputs and generation of steering commands by the distinct steering circuitry (Cregg and others 2020; Usseglio and others 2020) are still possible during tonic stimulation of Vglut2^+^ CnF neurons. Panels A–I adapted from van der Zouwen and others (2021). FL, forelimb; HL, hindlimb; MTP, metatarsophalangeal.

Optogenetic activation of CnF Vglut2^+^ neurons controlled the initiation of locomotion and all locomotor speeds in mice, and this effect was robust from laboratory to laboratory (Caggiano and others 2018; Dautan and others 2021; Fougère and others 2021; Josset and others 2018; van der Zouwen and others 2021; Masini and Kiehn 2022; Figure 3A and B). During optogenetic experiments, an increase in laser power or stimulation frequency increased locomotor speed or elicited transitions between slow gaits (walking, trotting) and fast gaits (gallop, bound) in a linear corridor (Caggiano and others 2018), a motorized treadmill (Josset and others 2018), or an open field (Dautan and others 2021; Fougère and others 2021; van der Zouwen and others 2021; Figure 3C). Importantly, the footfall pattern and the limb kinematics evoked by stimulation of CnF Vglut2^+^ neurons were very close to those recorded in the same animals during spontaneous locomotion (van der Zouwen and others 2021; Figure 3D and E). Optogenetic stimulation of CnF Vglut2^+^ neurons did not prevent mice from slowing down and turning when approaching a wall in an open field (van der Zouwen and others 2021; Figure 3G–I). The motor signature of the slowing down during turning was close to the one reported when stimulating the steering circuitry, which comprises reticular V2a neurons that receive visual information from the superior colliculus, a direct target of retinal ganglion cells (Bouvier and others 2015; Cregg and others 2020; Usseglio and others 2020). This indicates that during stimulation of CnF Vglut2^+^ neurons, integration of visual inputs is still possible, ensuring adaptable navigation (van der Zouwen and others 2021). This is consistent with the ability of mice to navigate in a hole board during CnF Vglut2^+^ optogenetic stimulation (Caggiano and others 2018).

### Inhibition

Optogenetic inhibition of CnF Vglut2^+^ neurons slowed down ongoing locomotion on a motorized treadmill (Josset and others 2018). Another team reported that optogenetic inhibition of MLR Vglut2^+^ neurons impeded running, although the distinction between CnF and PPN was not done (Roseberry and others 2016). Chemogenetic inhibition of CnF Vglut2^+^ neurons decreased the ability of mice to use the fastest gaits (Caggiano and others 2018), reduced locomotor speed during escape, and lowered maximal acceleration without affecting the use of slower speed ranges during exploratory locomotion in an open field (Masini and Kiehn 2022). Chemogenetic inhibition of CnF Vglut2^+^ neurons did not prevent the prolocomotor effects of chemogenetic activation of caudal PPN Vglut2^+^ neurons (Masini and Kiehn 2022).

### Activity during Behavior

There was a positive correlation between locomotor speed and the firing frequency of some mouse CnF Vglut2^+^ neurons recorded extracellularly during long bouts of locomotion (30–60 s) on a motorized treadmill (Caggiano and others 2018). However, only a fraction of MLR neurons encoded locomotion speed overall (for review, Arber and Costa 2022; Caggiano and others 2018; Carvalho and others 2020; Ferreira-Pinto and others 2021; Roseberry and others 2016).

### Cell Properties

CnF Vglut2^+^ neurons have homogeneous electrophysiologic properties (Dautan and others 2021). Most are fast-adapting neurons, lack persistent sodium currents, and display weak-amplitude, tetrodotoxin-resistant oscillating properties of their membrane potential in the 20- to 40-Hz range. CnF Vglut2^+^ neurons have a more complex dendritic structure than PPN Vglut2^+^ ones (Dautan and others 2021).

### Target-Specific Effects

**Figure 4.**
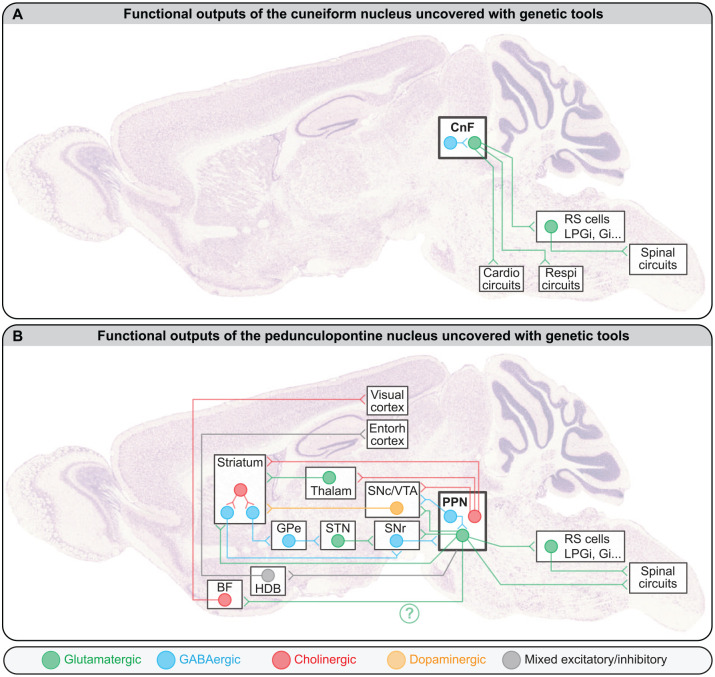
Functional outputs of (A) the cuneiform nucleus (CnF) and (B) the pedunculopontine nucleus (PPN). Note that only the projections tested functionally with genetic tools on rodents were included here, but more anatomical projections were reported. For instance, the descending cholinergic projections of the PPN are well established (Gut and Mena-Segovia 2019; Mena-Segovia and Bolam 2017; for review, Ryczko and Dubuc 2013). For the sake of simplicity, in the CnF and PPN, all cells with the same transmitter were pooled in a single circle. The interconnectivity among mesencephalic locomotor region cell types and whether the different projection patterns originate from distinct populations of neurons are not fully resolved. The green question mark highlights that it is not known whether this CamKIIa^+^ projection to BF originates from PPN or CnF, because the distinction was not made in Lee and others (2014). For details about the behavioral role of these projections, see main text. BF, basal forebrain; Cardio, cardiovascular; Entorh, entorhinal; Gi, gigantocellular nucleus; GPe external globus pallidus; HDB, horizontal limb of the diagonal band of Broca; LPGi, lateral paragigantocellular nucleus; Respi, respiratory; RS, reticulospinal; SNc, dopaminergic neurons of the substantia nigra pars compacta; SNr, substantia nigra pars reticulata; STN, subthalamic nucleus; Thalam, thalamus; VTA, dopaminergic neurons of the ventral tegmental area. The brain picture was kindly provided by GENSAT and previously used as a background image in Fougère and others (2019).

CnF Vglut2^+^ neurons control locomotion through projections to multiple reticular nuclei (Figure 4A), including the LPGi, gigantocellular nucleus (Gi), gigantocellular reticular nucleus alpha part (GiA), gigantocellular reticular nucleus ventral part (GiV), and caudal raphe nuclei, which all contain reticulospinal neurons (Bretzner and Brownstone 2013; Caggiano and others 2018; Capelli and others 2017), although the CnF/PPN distinction was not systematically made (for review, Arber and Costa 2022; Leiras and others 2022). Optogenetic stimulation of the Vglut2^+^ terminals originating from the MLR and targeting the LPGi evoked locomotion in mice (Capelli and others 2017). Vglut2^+^ neurons retrogradely labeled in the MLR from the LGPi evoked locomotion when optogenetically stimulated, and most of these neurons resided in the CnF (Capelli and others 2017). CnF projections to other reticular nuclei are likely functional, since CnF electrical stimulation evoked excitatory responses in gigantocellular nucleus V2a neurons recorded via calcium imaging in mice (Bretzner and Brownstone 2013).

Beyond locomotion, CnF glutamatergic neuron activation increased respiration in mice (Hérent and others 2021). CnF Vglut2^+^ neurons project to the pre-Bötzinger complex, where part of the respiratory central pattern generator resides (Hérent and others 2021). Optogenetic stimulation of CnF Vglut2^+^ neurons increases the respiratory rhythm, even in the absence of locomotion (Hérent and others 2021). These projections are likely phylogenetically old, since in lamprey, glutamatergic projections originating from the dorsal part of the MLR (the possible homolog of the CnF) increased respiration (Gariépy and others 2012). The CnF also regulates cardiovascular activity (for review, Noga and Whelan 2022; Ryczko and Dubuc 2013). DBS of the MLR increases heart rate, and this seems to be an indicator of successful CnF targeting (rat, Korte and others 1992; cat, Opris and others 2019; pig, Chang, Santamaria, and others 2021). The pathway involved likely comprises projections from the CnF to the LPGi; these would send input to raphe magnus 5-HT neurons, which modulate the nucleus of the solitary tract in mice (Netzer and Sevoz-Couche 2021).

### Stimulation in a PD Model

**Figure 5.**
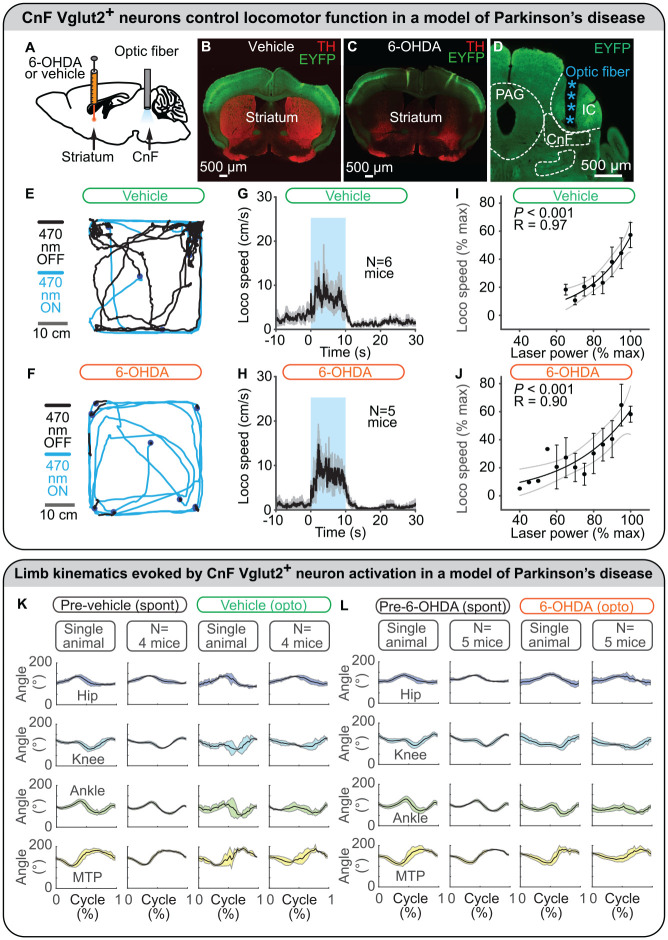
Optogenetic stimulation of glutamatergic neurons in the cuneiform nucleus (CnF) controls locomotion in a mouse model of Parkinson disease. (A–D) To model Parkinson disease, a neurotoxin (6-hydroxydopamine [6-OHDA]) was injected in the striatum of mice expressing the ion-sensitive channel channelrhodopsin and the fluorescent protein EYFP in glutamatergic neurons (positive for the vesicular glutamatergic transporter 2 [Vglut2]). In control mice, only a vehicle solution was injected in the striatum. Intrastriatal 6-OHDA destroyed dopaminergic fibers positive for tyrosine hydroxylase in the striatum, but intrastriatal vehicle solution did not. An optic fiber was implanted in the CnF and connected to a laser-generating blue light (470 nm) to activate CnF Vglut2^+^ neurons. (E, F) In an open field, mouse locomotion was filmed from above during optogenetic stimulation (10 s, blue lines) and in between stimulations (80 s, black lines). Between optogenetic stimulations, spontaneous locomotion was severely lowered in 6-OHDA mice as compared with control mice, in which dopaminergic neurons were not destroyed. (G, H) Optogenetic stimulation of CnF Vglut2^+^ neurons increased locomotor speed (mean ± SEM) in 6-OHDA mice as in control mice. (I, J) Increasing laser power increased locomotor speed in 6-OHDA mice as in control mice. Data are presented as mean ± SEM. Black line, sigmoidal fit; dotted lines, 95% CI. (K, L) In a linear corridor, hindlimb kinematics were filmed from the side and analyzed with DeepLabCut (see also Figure 3E). The angle excursions (mean ± SD) in the hip, knee, ankle, and metatarsophalangeal (MTP) joints during locomotion evoked by optogenetic stimulation of CnF Vglut2^+^ neurons were largely similar to those recorded during spontaneous locomotion before the destruction of dopaminergic neurons. In control mice, hindlimb joint angle excursions (mean ± SD) during locomotion evoked by optogenetic stimulation of CnF Vglut2^+^ neurons were largely similar to those recorded during spontaneous locomotion before the injection of a vehicle solution in the striatum. Panels A–L adapted from Fougère and others (2021). IC, inferior colliculus; PAG, periaqueductal gray.

In mice, intrastriatal injections of the neurotoxin 6-hydroxydopamine (6-OHDA) destroyed dopaminergic neurons (Figure 5A–C) and decreased spontaneous locomotion (Figure 5E and F; Fougère and others 2021). Although 6-OHDA does not mimic the chronic aspect of the degeneration observed in PD, it is useful for the preclinical evaluation of potential therapeutic interventions (Duty and Jenner 2011). In this type of PD model, optogenetic stimulation of CnF Vglut2^+^ neurons increases the time spent in locomotion, the number of locomotor initiations, and the locomotor speed (Figure 5E–H). The hindlimb joint angular excursions are largely similar to those recorded before the 6-OHDA lesion (Figure 5K and L). The level of CnF Vglut2^+^ neuron activation positively controls locomotor speed in such PD conditions (Figure 5I and J), and mice can slow down and turn when approaching a wall of the open field (Figure 5E and F; Fougère and others 2021) as in intact mice (van der Zouwen and others 2021).

**Figure 6.**
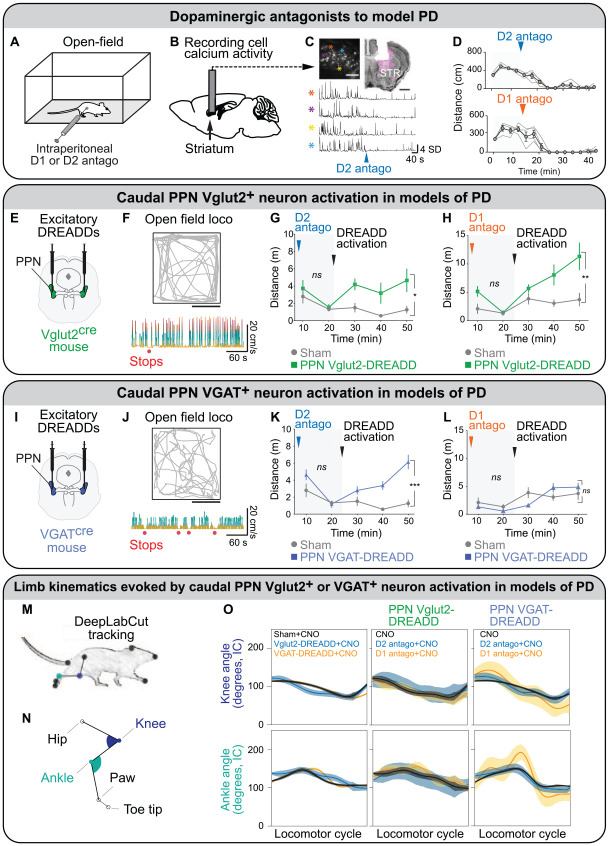
Chemogenetic activation of glutamatergic (Vglut2^+^) or GABAergic (VGAT^+^) neurons in the caudal pedunculopontine nucleus has different effects on locomotion in mouse models of Parkinson disease (PD). (A) To model the akinesia seen in PD, Masini and Kiehn (2022) injected intraperitoneally in mice a D2 antagonist (haloperidol) or a D1 antagonist (SCH 23390). (B) Calcium activity of striatal neurons were measured with a miniaturized microscope coupled with a GRIN lens positioned above the dorsal striatum (STR) of D1-cre mice injected in the STR with an adeno-associated virus expressing the genetically encoded calcium sensor GCaMP6s in a cre-dependent manner. (C) Top left: field of view showing neurons expressing GCaMP6s in white (scale 100 μm). Top right: coronal section illustrating lens position above the dorsal STR (scale 1 mm), with GCamP6s in magenta and neurotrace in black. The lower panel presents a typical example of calcium activity (*Z* score) from four cells in the field of view after intraperitoneal application of a D2 antagonist (haloperidol). (D) Distance moved in the open field before and after the dopaminergic antagonist injection. Individuals are represented by thin lines; group mean ± SEM are in black. (E, I) Viral strategies to bilaterally express excitatory DREADD in caudal pedunculopontine nucleus (PPN) Vglut2^+^ neurons or caudal PPN VGAT^+^ neurons. (F, J) Top: representative trajectory of the animal in the open field (scale, 25 cm) after DREADD activation. Bottom: instantaneous speed of the animal following DREADD activation in PPN Vglut2^+^ or VGAT^+^ neurons. Stop events in absence of in-place behavior are illustrated by red dots. (G, H, K, L) Timeline effect of D1 or D2 antagonist before (light gray background) and after (gray) DREADD activation in sham. Vglut2-cre mice injected in the caudal PPN with DREADD (green) and VGAT-cre mice injected in the caudal PPN with DREADD (blue). Two-way repeated measures analysis of variance with graph reporting group effect and analysis done for each period (i.e., before and after DREADD activation). Data are presented as mean ± SEM. ns, not significant. *P < 0.05. **P < 0.01. ***P < 0.001. (M, N) Hindlimb kinematics were filmed from the side, and the angular excursions in the knee and ankle joints were analyzed with DeepLabCut. (O) Angular excursions (mean ± 95% CI) of knee and ankle joints during a complete locomotor cycle. Panel A adapted from van der Zouwen and others (2021). Panel B adapted from Fougère and others (2021). Panels C–O adapted from Masini and Kiehn (2022). CNO, clozapine N-oxide; DREADD, designer receptors exclusively activated by designer drugs.

In another mouse PD model based on systemic injections of dopaminergic antagonists, chemogenetic activation of CnF Vglut2^+^ neurons increased locomotor activity (Masini and Kiehn 2022). Evoked behavior consists of succession of high-speed locomotion and long stops, reminiscent of a “darting” behavior, which is displayed when animals try to avoid a predator (Masini and Kiehn 2022). Such behavior is consistent with the inputs to the CnF, which are associated with defensive behavior (Caggiano and others 2018; Dautan and others 2021; for review, Leiras and others 2022; Ryczko and Dubuc 2013). In contrast, chemogenetic stimulation of PPN Vglut2^+^ neurons produced more continuous slow-speed locomotion in the same PD model (Figure 6; Masini and Kiehn 2022; see Stimulation in a PD Model in PPN Vglut2^+^ Neurons section).

Altogether this indicates that CnF Vglut2^+^ neurons are a relevant target to improve locomotor function and control locomotor speed, without disrupting the ability to slow down and turn during navigation in PD conditions. Vglut2^+^ neurons are present in the human MLR according to immunohistochemistry and in situ hybridization, but their degeneration status in PD needs further investigation (Sébille and others 2019). CnF Vglut2^+^ neurons were recently identified as a relevant target to improve locomotor function after partial spinal cord injury in mice (Roussel and others 2022).

## CnF VGAT Neurons: Locomotor Arrests

Optogenetic stimulation of CnF VGAT^+^ neurons stopped ongoing locomotion in mice walking on a motorized treadmill (Caggiano and others 2018). In line with this, optogenetic activation of MLR VGAT^+^ neurons decreased walking speed on a trackball in mice, although the distinction between CnF and PPN was not done (Roseberry and others 2016). In vivo extracellular recordings showed that optogenetic stimulation of MLR VGAT^+^ neurons reduced the spiking of non-GABAergic (thus possibly glutamatergic) neurons (Roseberry and others 2016). Although the selective manipulation of CnF VGAT^+^ neurons was not tested in an animal of PD, it is likely that activation of these neurons should worsen the locomotor deficits.

## PPN Vglut2^+^ Neurons: Slow Gaits, Posture Adjustments, and Locomotor Arrests

### Stimulation

As compared with the CnF, PPN neurons have a more complex projection pattern (Figure 4B). More variable outcomes were reported when PPN neurons were stimulated. PPN Vglut2^+^ neurons were first noted to evoke slow locomotor gaits in a linear corridor (walking and trotting) but not fast gaits (Caggiano and others 2018). Higher intensity of photostimulation of PPN Vglut2^+^ led to higher speed (Caggiano and others 2018). Chemogenetic activation of the caudal PPN Vglut2^+^ increased low-speed locomotor activity in an open field and reduced the number of stops per time unit relative to the control condition (Masini and Kiehn 2022). PPN Vglut2^+^ neurons stimulation also increased exploratory locomotion in the hole board test, in which exploration was motivated by olfactory cues from conspecifics (Caggiano and others 2018). Interestingly, environment scanning during locomotion was occasionally cited during electrical stimulation of the MLR in pigs (Chang, Santamaria, and others 2021). Another team reported that chemogenetic activation of PPN Vglut2^+^ neurons promoted wakefulness and mild anxiety, and this translated to increased locomotor activity on a running wheel placed in mouse home cages during daytime (i.e., when mice are supposed to be quieter and are about to sleep; Kroeger and others 2017).

Activation of PPN Vglut2^+^ neurons can also stop locomotion or have no effect on locomotor activity. A first team reported that optogenetic stimulation of PPN Vglut2^+^ neurons did not initiate locomotion but slowed down or stopped ongoing locomotion on a motorized treadmill in mice (Josset and others 2018). A second team showed that optogenetic activation of PPN Vglut2^+^ neurons did not initiate locomotion, slowed down ongoing locomotion on a motorized treadmill, and reduced the distance traveled in an open field (Dautan and others 2021). When mice were placed on an elevated grid, optogenetic activation of PPN Vglut2^+^ neurons reduced the distance traveled, decreased the number of rearing events, and increased foot slips (Dautan and others 2021). A third team indicated that in rats optogenetic stimulation of PPN CamKIIa^+^ neurons (among which most but not all are glutamatergic; Kreeger and others 2021; Meng and others 2016; Roseberry and others 2016; Wang and others 2013) increased locomotion in 76% of animals but slowed down or stopped locomotion in 24% of animals, thus somehow recapitulating the variability of behavioral outcomes (Carvalho and others 2020). Altogether, this indicates that multiple groups of Vglut2^+^ neurons are present in the PPN and they likely control different aspects of motor behavior. Targeting Vglut2^+^ neurons in the caudal PPN is important to evoke prolocomotor effects (Figure 6; Caggiano and others 2018; for review, Leiras and others 2022; Masini and Kiehn 2022).

### Inhibition

Optogenetic inhibition of PPN Vglut2^+^ neurons stopped locomotion in most trials or slowed down locomotion by increasing hindlimb extensor activity on a motorized treadmill in mice (Josset and others 2018). Optogenetic inhibition of PPN Vglut2^+^ neurons did not increase the distance traveled in an open field and did not decrease foot slips in the elevated grid (Dautan and others 2021). CnF Vglut2^+^ neurons can evoke all gaits independently of PPN Vglut2^+^ neurons: when PPN Vglut2^+^ neurons are inhibited through chemogenetics, optogenetic stimulation of CnF Vglut2^+^ neurons can still evoke all gaits, including gallop and bound, nevertheless with a reduction in the maximal locomotor speed (Caggiano and others 2018).

### Activity during Behavior

During long bouts of locomotion on a motorized treadmill (30–60 s), 1) some PPN Vglut2^+^ were activated at the beginning of the locomotor bout and decreased their activity thereafter; 2) some showed no correlation with locomotor speed; and 3) some showed a correlation with locomotor speed (Caggiano and others 2018; see also Roseberry and others 2016, although the CnF/PPN distinction was not done). Extracellular electrophysiological recordings of nonidentified PPN cells showed that 29% of neurons correlated positively and 17% negatively with locomotor speed. Overall, only a fraction of PPN cells correlated with locomotor speed (Carvalho and others 2020; Norton and others 2011). This is in line with recent work showing heterogeneity in the behavioral effects evoked by the stimulation of different cell types in the PPN: some PPN glutamatergic neurons projecting to the spinal cord encoded body rearing, and others projecting to an output station of the basal ganglia encoded forelimb movements such as handling and grooming (Ferreira-Pinto and others 2021).

### Cell Properties

PPN Vglut2^+^ neurons are more heterogeneous than CnF ones (Dautan and others 2021). Examination of their firing pattern indicated that 86% of CnF neurons are fast adapting, while in the PPN, 49% are fast adapting, 21% are slow adapting, and 30% are nonadapting (Dautan and others 2021). PPN Vglut2^+^ neurons display high-amplitude, tetrodotoxin-sensitive oscillations of their membrane potential in the 10- to 20-Hz range. Most PPN Vglut2^+^ neurons express persistent sodium currents, contrary to CnF ones. PPN Vglut2^+^ dendritic trees are less complex than CnF ones (Dautan and others 2021). Whether such variability goes hand in hand with the variety of projections and/or behavioral outcomes remains to be examined.

### Target-Specific Effects

It is likely that the diversity of behavioral outcomes occurring when stimulating PPN Vglut2^+^ neurons involves different projection neurons (Figure 4B).

A first set of projections from PPN Vglut2^+^ neurons provided descending input to several reticular nuclei (Caggiano and others 2018) that contain different reticulospinal neurons able to shape locomotor pattern and rhythm in the freely behaving mouse (Lemieux and Bretzner 2019; Figure 4B). Future studies should determine whether PPN Vglut2^+^ neurons segregate to either target V2a neurons responsible for locomotor stops (Bouvier and others 2015; Cregg and others 2020; Usseglio and others 2020) or reticulospinal neurons in the LPGi that convey the locomotor drive to the spinal cord (Capelli and others 2017). This is possible since in zebrafish, electrical stimulation of the MLR activated diverse populations of V2a reticulospinal neurons (Carbo-Tano and others 2022). In lamprey, reticulospinal “stop cells” (Juvin and others 2016) were under control of direct glutamatergic input from the MLR (Grätsch and others 2019).

A second projection from PPN Vglut2^+^ neurons targets the striatum (Figure 4B), avoids spiny projection neurons, and directly connects striatal cholinergic interneurons and GABAergic fast-spiking interneurons (Assous and others 2019). Unilateral activation of PPN Vglut2^+^ results in disynaptic inhibition of medium spiny neurons and evokes head turns ipsilaterally to PPN stimulation.

A third projection of PPN Vglut2^+^ neurons targets dopaminergic neurons of the substantia nigra pars compacta (SNc; Figure 4B). Optogenetic stimulation of these axons evoked spiking in SNc dopaminergic neurons (Galtieri and others 2017). This pathway is well positioned to increase locomotor output since activation of SNc dopaminergic neurons increases the vigor of future movement (da Silva and others 2018; Howe and Dombeck 2016).

A fourth projection of PPN Vglut2^+^ neurons targets dopaminergic neurons of the ventral tegmental area (VTA; Figure 4B). Optogenetic stimulation of this pathway promoted behavioral reinforcement in an intracranial self-stimulation task, during which the animal could introduce its nose in a hole to trigger optogenetic stimulation of PPN Vglut2^+^ terminals in the VTA (Yoo and others 2017).

A fifth projection from PPN Vglut2^+^ neurons targets the spinal cord and encodes rearing in mice. Its activation induced body stretching whereas deactivation shortened body length (Ferreira-Pinto and others 2021).

A sixth projection of likely Vglut2^+^ neurons located in the PPN and adjacent mesencephalic reticular formation targets the substantia nigra pars reticulata (Ferreira-Pinto and others 2021), an output station of the basal ganglia that inhibits the MLR (Roseberry and others 2016; Figure 4B). These neurons express the genetic marker Rbp4, are ChAT^–^ and VGAT^–^ (thus likely Vglut2^+^), and their activation terminated locomotion (Ferreira-Pinto and others 2021).

A possible seventh projection of PPN CamKIIa^+^ neurons (among which most but not all are glutamatergic; Kreeger and others 2021; Meng and others 2016; Roseberry and others 2016; Wang and others 2013) would target the horizontal limb of the diagonal band of Broca in the basal forebrain, which sends input to the entorhinal cortex (Carvalho and others 2020; Figure 4B). This pathway likely provides speed-related information to update the representation of the animal’s position in the environment during navigation. Future studies should identify whether a mix of Vglut2^+^ and VGAT^+^ PPN neurons is involved because ascending PPN inputs produce excitation and inhibition in the basal forebrain (Carvalho and others 2020).

A possible eighth projection of PPN CamKIIa^+^ neurons would target cholinergic neurons in the basal forebrain that send input to the visual cortex (Lee and others 2014; Figure 4B). Activation of these CamKIIa^+^ neurons increases the gain of cortical cells responding to visual stimuli. Future studies should identify whether PPN and/or CnF Vglut2^+^ neurons are involved because the distinction was not made (Lee and others 2014).

### Stimulation in a PD Model

In mouse models of PD based on systemic injection of dopaminergic antagonists (Figure 6A–D), optogenetic or chemogenetic stimulation of Vglut2^+^ neurons in the caudal PPN increased slow exploratory locomotion in an open field (Masini and Kiehn 2022; Figure 6E–H). The limb kinematics evoked by such stimulations were largely normal when angular excursions in the knee and ankle were measured (Figure 6M–O). Mice could climb stairs or avoid pillars on the way, showing that activation of caudal PPN Vglut2^+^ neuron allows for adaptable navigation, as CnF Vglut2^+^ neurons do (Fougère and others 2021; van der Zouwen and others 2021). The prolocomotor effect of caudal PPN Vglut2^+^ neurons was independent of CnF Vglut2^+^ neurons that were deactivated through chemogenetics in those experiments (Masini and Kiehn 2022). This indicates that a subtype of PPN Vglut2^+^ neurons constitutes a relevant target to improve locomotor function in PD conditions. Further investigation is needed to understand which PPN cell subtype promotes locomotion and which inhibits locomotion. The superiority of the caudal PPN over the rostral PPN as a target to evoke locomotion is consistent with previous literature. In parkinsonian rats, DBS of the anterior PPN worsened gait, whereas DBS of the caudal PPN improved gait (Gut and Winn 2015). This is consistent with the rostral PPN containing more GABA neurons projecting to the basal ganglia and the caudal PPN containing more glutamatergic neurons projecting to reticular nuclei (for review, Martinez-Gonzalez and others 2011; Mena-Segovia and Bolam 2017; Ryczko and Dubuc 2013).

## PPN GABAergic Neurons: Locomotor Arrests and Slow Segmented Locomotion

### Stimulation

As for PPN Vglut2^+^ neurons, there is some variability in the locomotor effects evoked by activation of PPN VGAT^+^ neurons, and this may be linked to the diverse targets of these neurons (Figure 4B). The duration of the stimulation train can produce different behavioral outcomes. Relatively short optogenetic activation of PPN VGAT^+^ neurons (2-s trains) did not evoke locomotion and slowed down ongoing locomotion of mice in a linear corridor (Caggiano and others 2018). In line with this, locomotor speed was decreased by optogenetic activation (5-s trains) of MLR VGAT^+^ neurons in mice walking on a trackball, although the difference between PPN and CnF was not made (Roseberry and others 2016). In contrast, longer periods of optogenetic stimulation (10-s trains) or chemogenetic activation of PPN VGAT^+^ neurons could initiate locomotion and evoke a modest increase in the distance traveled in an open field, but the locomotor bouts were interrupted by abundant short stops (Masini and Kiehn 2022). The decrease in locomotion evoked by PPN VGAT^+^ neurons reported by Caggiano and colleagues (2018) may have involved local projections to PPN Vglut2^+^ neurons (Roseberry and others 2016), whereas the prolocomotor effect reported by Masini and Kiehn (2022) could have involved ascending inhibition of the subthalamic nucleus (Guillaumin and others 2021; Schweizer and others 2014), which sends excitatory input to the output stations of the basal ganglia that inhibit PPN Vglut2^+^ neurons (Masini and Kiehn 2022).

### Target-Specific Effects

PPN VGAT^+^ neurons send ascending projections to SNc dopaminergic neurons and, to a lesser extent, to VTA (Gut and others 2022; Figure 4B). Optogenetic stimulation of PPN VGAT^+^ inputs to SNc decreased striatal dopamine release, reduced the distance traveled in the open field, and increased rearing, but it did not prevent the animal from doing other behaviors, such as rotarod locomotion, pasta handling and eating, or aversive conditioning (Gut and others 2022). However, activation of PPN VGAT^+^ projections to SNc interfered with the initiation of a prelearned motor sequence (Gut and others 2022).

### Stimulation in a PD Model

Chemogenetic or optogenetic stimulation of caudal caudal PPN VGAT^+^ neurons could increase locomotor activity in mice made parkinsonian by systemic injection of a D2 dopaminergic antagonist but not in mice made parkinsonian with a D1 antagonist (Figure 6I–L; Masini and Kiehn 2022). The prolocomotor effect is less strong than when caudal PPN Vglut2^+^ neurons or CnF Vglut2^+^ neurons are stimulated (Masini and Kiehn 2022). Caudal PPN VGAT^+^ neuron activation induced rather abnormal limb movements, such as longer stance phase and limb joint hyperextension (Figure 6M–O), and reduced hindlimb-forelimb coordination during the ladder test (Masini and Kiehn 2022). Altogether, PPN VGAT^+^ neurons are likely not the best neurons to stimulate to improve locomotor function in PD conditions, since they either stop locomotion or evoke segmented locomotor bouts, and the evoked limb movements are not normal.

## PPN Cholinergic Neurons: A Complex Role

### Stimulation

The role of PPN cholinergic neurons is still unresolved, maybe because these cells have diverse and complex projection patterns as well (for review, Mena-Segovia and Bolam 2017; Figure 4B). Optogenetic stimulation of PPN ChAT^+^ neurons slightly increased the speed of ongoing locomotion, but such effect was much weaker than that evoked by MLR Vglut2^+^ neurons (Roseberry and others 2016). In contrast, optogenetic stimulation of PPN ChAT^+^ neurons slightly decreased ongoing locomotor speed in a linear corridor in mice (Caggiano and others 2018) or slowed down locomotion by increasing motor tone in hindlimb extensors, thus increasing stance phase duration (Josset and others 2018). In the three aforementioned studies, optogenetic activation did not initiate locomotion. Chemogenetic activation of PPN ChAT^+^ neurons did not affect the distance traveled in an open field in mice (Ruan and others 2022). This is in contrast with the prolocomotor effect of PPN ChAT^+^ neurons noted in PD conditions in rats (see Stimulation in a PD Model in the PPN Cholinergic Neurons section; Pienaar, Gartside, and others 2015; Sharma and others 2020).

### Inhibition

V﻿ariable results were reported. Optogenetic inhibition of PPN ChAT^+^ neurons decreased spontaneous locomotion in the open field in rats (Xiao and others 2016). Optogenetic inhibition of PPN ChAT^+^ neurons slowed down by increasing extensor burst duration, and this slowing down effect was surprisingly similar to that resulting from their optogenetic activation (Josset and others 2018). Chemogenetic inhibition of PPN ChAT^+^ neurons did not change the distance traveled in an open field in rats (Dautan and others 2020) and mice (Ruan and others 2022).

### Lesion

The effect of lesioning PPN cholinergic neurons in rats depends on the task. It was reported to impair performance in complex motor tasks such as the accelerating rotarod (MacLaren and others 2014, Xiao and others 2017). Destruction of PPN cholinergic neurons in rats also increased the paw print area, the speed at which the paw transitioned to next floor contact (suggesting postural instability), and freezing in rats walking on a catwalk (Chambers and others 2021). In contrast, another team indicated that PPN cholinergic lesion did not induce gait disturbance in the CatWalk test but aggravated the locomotor deficits resulting from a lesion of dopaminergic neurons (i.e., increased the number of freezing of gaits and limb dual stance; Xiao and others 2017). In humans with PD, loss of MLR cholinergic neurons was associated with falls, and in monkeys, destruction of PPN cholinergic neurons reproduced the locomotor deficits observed in a PD model based on the destruction of dopaminergic neurons through intoxication with MPTP (Karachi and others 2010).

### Activity during Behavior

In vivo extracellular recordings showed that PPN cholinergic neurons changed their activity pattern in relation with brain state transitions, in particular to changes in cortical activity (Mena-Segovia and others 2008). Their activity increased after a pinch of the hindpaw that increased cortical activity, and PPN ChAT^+^ neuron activation preceded cortical activation (for review, Mena-Segovia and Bolam 2017; Petzold and others 2015). PPN cholinergic neurons also responded to rewards, and these responses were influenced by rule switching in the behavioral task (Ruan and others 2022).

### Cell Properties

In mice, the rostrocaudal location of the PPN ChAT^+^ neurons correlated with their electrophysiologic properties. Neurons that have high threshold membrane potential oscillations with high frequency (β-γ range, 12–80 Hz) and low power are in the caudal PPN. Neurons that display low-frequency oscillations (α range, 8–12 Hz) and high power are in the rostral PPN. Among neurons displaying a transient outward potassium current (A-current), early- and late-firing neurons could be distinguished, with late-firing ones being more in the caudal PPN (Baksa and others 2019). These cell groups are consistent with those recorded in rats (Mena-Segovia and others 2008; for review, Mena-Segovia and Bolam 2017). Whether these different properties are linked with different projection patterns or behavioral outcomes remains to be examined.

### Target-Specific Effects

Here I focus on PPN projections recently tested functionally with genetic tools in rodents (Figure 4B), but additional projections to the basal ganglia or downstream to reticular nuclei are well established (Gut and Mena-Segovia 2019; Mena-Segovia and Bolam 2017; for review, Ryczko and Dubuc 2013). A first target of PPN cholinergic neurons is the striatum (Brimblecombe and others 2018; Dautan and others 2014; Dautan and others 2020; Klug and others 2018), and activation of these ascending cholinergic fibers increased firing in striatal cholinergic interneurons and inhibited spiny projection neurons (Dautan and others 2020). Chemogenetic inhibition of PPN ChAT^+^ projections to the striatum did not change locomotor activity in the open field (Dautan and others 2020).

A second target of PPN ChAT^+^ neurons are mesodiencephalic dopaminergic neurons (SNc/VTA; Figure 4B). The locomotor role of this ascending input is not resolved. Optogenetic activation of PPN cholinergic projections to VTA either increased (Dautan and others 2016) or decreased (Xiao and others 2016) locomotor activity in an open field. Such stimulation was associated with excitation of dopaminergic neurons in rats (Dautan and others 2016). Optogenetic activation of ascending PPN ChAT^+^ projections to SNc increased locomotor activity in an open field, and their inhibition decreased locomotion (Xiao and others 2016). In contrast, another team reported that optogenetic activation of the ascending PPN ChAT^+^ projections to A9 did not modify the distance traveled or locomotor speed in an open field in mice (Ruan and others 2022). The reason for such discrepancy is unclear.

A third target of PPN cholinergic neurons is the thalamus (Huerta-Ocampo and others 2020; Figure 4B). Optogenetic stimulation of the ascending projections of PPN ChAT^+^ neurons to either the mediodorsal thalamic nucleus or the parafascicular thalamus nucleus did not modify the distance traveled or the locomotor speed in an open field (Ruan and others 2022). This ascending PPN ChAT^+^ inputs to thalamus likely play a role in nonlocomotor aspects such as control of sleep or arousal (Gut and Mena-Segovia 2019; Mena-Segovia and Bolam 2017; Ni and others 2016) or in cognitive flexibility such as reversal learning (Ruan and others 2022).

### Stimulation in a PD Model

Chemogenetic stimulation of PPN ChAT^+^ neurons improved locomotor performance in a rat model of PD based on injection in SNc of the proteasomal inhibitor lastacystin, which resulted in a 48% loss of dopaminergic SNc neurons and 61% loss of PPN cholinergic neurons (Pienaar, Gartside, and others 2015; Pienaar, Harrison, and others 2015). Chemogenetic activation of PPN ChAT^+^ neurons increased the latency to fall on the accelerating rotarod, the stepping distance, and the number of locomotor bouts in an open field in rats (Sharma and others 2020). These effects could involve striatal circuits, since such stimulation increases in striatal interneurons the expression of c-fos, a marker of cell activation (Sharma and others 2020). The prolocomotor effect is also consistent with the observation that loss of cholinergic PPN neurons correlated with locomotor deficits in patients with PD (Karachi and others 2010). The prolocomotor effect could involve a descending cholinergic drive from PPN to reticulospinal neurons as shown in lamprey, in which the cholinergic component of the MLR was found to send direct excitation to reticulospinal neurons through nicotinic receptors (Le Ray and others 2003) and indirect descending excitation to reticulospinal neurons through a group of muscarinoceptive cells that in turn send excitation to reticulospinal cells (Smetana and others 2010). However, the prolocomotor effect evoked by chemogenetic activation of PPN cholinergic neurons in a rat model of PD (Sharma and others 2020) is in contrast with the modest or inhibitory effect on locomotion evoked by optogenetic or chemogenetic stimulation of PPN cholinergic neurons in intact rodents (Caggiano and others 2018; Josset and others 2018; Roseberry and others 2016). Whether PPN cholinergic neurons acquire a stronger modulatory role on locomotor output in PD conditions is not known. In patients with PD, cholinesterase inhibitors do not strongly improve locomotor function, although the interpretation relative to the PPN is limited since several regions are affected by such pharmacologic intervention (Mancini and others 2019).

Altogether, a subset of PPN ChAT^+^ neurons appears to be a relevant target in PD conditions to improve locomotor function. To identify the subtype of interest, future studies should determine whether different PPN cholinergic neurons control different aspects of locomotion through different brain targets (Figure 4B).

## Implications for Disease and Future Directions

The recent studies strengthened the idea that the MLR is a multifunctional brain region (Ryczko and Dubuc 2013) that comprises cell types with opposing effects on motor output (Arber and Costa 2022; for recent review, Ferreira-Pinto and others 2018; Leiras and others 2022). The best cell type to target in the MLR to improve locomotor function in PD is just starting to be studied in animal research. Most clinical studies have targeted the PPN, leaving the CnF largely unexplored (Chang and others 2020; Goetz and others 2019). The CnF is starting to be considered in some clinical studies aiming at reducing freezing of gait in PD. A nontrivial task for neurosurgeons is to differentiate between CnF and PPN when positioning DBS electrodes. To improve the precision of stereotaxic targeting, a team defined a Brainstem Normalized Coordinate System in relation to the pontomesencephalic junction and found that good DBS responders had their electrodes in a site that encompassed the posterior PPN and CnF (Goetz and others 2019). A prospective pilot trial from another team revealed encouraging results when targeting the CnF in a patient with PD and levodopa-resistant freezing of gait (improved “timed up and go” test, increased stride length and velocity, longer swing phase duration; Chang, Cajigas, and others 2021). Nevertheless, selectively targeting the CnF may not be sufficient to drastically change the behavioral outcome, because CnF glutamatergic and GABAergic cells are intermingled. A clinical study in which CnF DBS was performed in 5 patients reported increased swing phase duration and shorter anticipatory adjustments but no improvement in the clinical scores (Bourilhon and others 2022).

Stimulation interventions with more specific targeting are needed to improve their efficiency in PD. In animals, so far, the best cell types to activate to promote locomotion are CnF glutamatergic neurons (Fougère and others 2021), some glutamatergic neurons in the caudal PPN (Masini and Kiehn 2022), and some cholinergic PPN neurons (Pienaar, Gartside, and others 2015; Sharma and others 2020; Figure 2). The cells to avoid activating are the GABAergic neurons in the CnF (Caggiano and others 2018) and PPN, which stop locomotion (Caggiano and others 2018) or evoke slow segmented locomotion with abnormal limb movements (Masini and Kiehn 2022; Figures 2 and 6). Concerning PPN glutamatergic, cholinergic, and GABAergic neurons, further studies should differentiate those promoting locomotion from those stopping locomotion, although for PPN glutamatergic neurons, the caudal ones appear to be the best candidates to promote locomotion (Masini and Kiehn 2022). Another parameter to consider is the degeneration status of the target neurons. Cholinergic PPN neurons degenerate in PD (Sébille and others 2019), but whether CnF and PPN glutamatergic neurons degenerate in PD needs to be examined, since some noncholinergic neurons are lost in the MLR in PD (Sébille and others 2019).

Some of the controversies observed during manipulation or recording of MLR cell populations may also be related, at least in part, to differences in the behavioral approaches used to record locomotor activity. Head-fixed mice placed on a trackball can run continuously during 10 to 20 s (Roseberry and others 2016). On a treadmill, mice are made to walk during 30 to 50 s (Caggiano and others 2018). In contrast, in mice moving freely in an open field, the duration of a locomotor bout is ~1 s (Fougère and others 2021). Different network dynamics may be recruited in these substantially different locomotor behaviors. Moreover, some MLR cells may control movement in a context-dependent manner (Caggiano and others 2018; Gut and others 2022; Kim and others 2017). Such dependency was exemplified for dorsal raphe serotoninergic neurons: in the same mice, their optogenetic activation stopped locomotion in an open field but did not impair rotarod performance and did not stop locomotion in a linear corridor exposed to a bright light with water rewards at each end (Correia and others 2017).

Beyond PD, the MLR is considered a potential target in spinal cord injury (Bachmann and others 2013; Bonizzato and others 2021; Chari and others 2017; Hofer and others 2022; Richardson 2014; Stieglitz and others 2021), supranuclear palsy (Scelzo and others 2017; Leimbach and others 2019), primary progressive freezing of gait (Ostrem and others 2010; Wilcox and others 2011), and stroke (Fluri and others 2017). Selective activation of CnF glutamatergic neurons was recently shown to improve functional outcome in chronic spinal cord injury in mice (Roussel and others 2022). Better targeting of the cell types of interest should ideally be achieved in the future by using optimized DBS protocols, pharmacotherapy, or the development of optogenetics for human use (Ratner and others 2021). Optogenetic therapy is increasingly considered to improve visual function for diseases involving photoreceptor degeneration such as retinis pigmentosa. In nonhuman primates, retinal ganglion cells can be made to express ChrimsonR, an excitatory opsin sensitive to ~590-nm yellow light following adeno-associated virus injection in the retina (Gauvain and others 2021). The stable expression of this opsin allows reliable light-evoked spiking activity in retinal ganglion cells (Gauvain and others 2021). A first patient with retinis pigmentosa showed partial functional recovery after optogenetic therapy (Sahel and others 2021). Currently at least four clinical trials based on optogenetics aim to improve the visual function in patients with such neurodegenerative disease (De Silva and Moore 2022). This first breakthrough may lay the foundation for the future use of optogenetics to improve locomotor function in patients with PD, spinal cord injury, or other locomotor deficits.

## References

[bibr1-10738584221139136] Arber S Costa RM . 2022. Networking brainstem and basal ganglia circuits for movement. Nat Rev Neurosci 23:342–60. 10.1038/s41583-022-00581-w 35422525

[bibr2-10738584221139136] Assous M Dautan D Tepper JM Mena-Segovia J . 2019. Pedunculopontine glutamatergic neurons provide a novel source of feedforward inhibition in the striatum by selectively targeting interneurons. J Neurosci 39:4727–37. 10.1523/JNEUROSCI.2913-18.2019 PMC6561696 30952811

[bibr3-10738584221139136] Bachmann LC Matis A Lindau NT Felder P Gullo M Schwab ME . 2013. Deep brain stimulation of the midbrain locomotor region improves paretic hindlimb function after spinal cord injury in rats. Sci Transl Med 5:208ra146. 10.1126/scitranslmed.3005972 24154600

[bibr4-10738584221139136] Baksa B Kovács A Bayasgalan T Szentesi P Kőszeghy Á Szücs P , and others. 2019. Characterization of functional subgroups among genetically identified cholinergic neurons in the pedunculopontine nucleus. Cell Mol Life Sci 76:2799–815. 10.1007/s00018-019-03025-4 PMC6588655 30734834

[bibr5-10738584221139136] Bernau NA Puzdrowski RL Leonard RB . 1991. Identification of the midbrain locomotor region and its relation to descending locomotor pathways in the Atlantic stingray, *Dasyatis sabina*. Brain Res 557:83–94. 1747771 10.1016/0006-8993(91)90119-g

[bibr6-10738584221139136] Bonizzato M James ND Pidpruzhnykova G Pavlova N Shkorbatova P Baud L , and others. 2021. Multi-pronged neuromodulation intervention engages the residual motor circuitry to facilitate walking in a rat model of spinal cord injury. Nat Commun 12:1925. 33771986 10.1038/s41467-021-22137-9 PMC7997909

[bibr7-10738584221139136] Bourilhon J Mullie Y Olivier C Cherif S Belaid H Grabli D , and others. 2022. Stimulation of the pedunculopontine and cuneiform nuclei for freezing of gait and falls in Parkinson disease: cross-over single-blinded study and long-term follow-up. Parkinsonism Relat Disord 96:13–17. 35121249 10.1016/j.parkreldis.2022.01.010

[bibr8-10738584221139136] Bouvier J Caggiano V Leiras R Caldeira V Bellardita C Balueva K , and others. 2015. Descending command neurons in the brainstem that halt locomotion. Cell 163:1191–203. 10.1016/j.cell.2015.10.074 PMC4899047 26590422

[bibr9-10738584221139136] Bretzner F Brownstone RM . 2013. Lhx3-Chx10 reticulospinal neurons in locomotor circuits. J Neurosci 33:14681–92. 10.1523/JNEUROSCI.5231-12.2013 PMC6705172 24027269

[bibr10-10738584221139136] Brimblecombe KR Threlfell S Dautan D Kosillo P Mena-Segovia J Cragg SJ . 2018. Targeted activation of cholinergic interneurons accounts for the modulation of dopamine by striatal nicotinic receptors. eNeuro 5. https://www.eneuro.org/content/5/5/ENEURO.0397-17.2018 10.1523/ENEURO.0397-17.2018 PMC6220583 30406189

[bibr11-10738584221139136] Brocard F Dubuc R . 2003. Differential contribution of reticulospinal cells to the control of locomotion induced by the mesencephalic locomotor region. J Neurophysiol 90:1714–27. 10.1152/jn.00202.2003 12736238

[bibr12-10738584221139136] Brocard F Ryczko D Fénelon K Hatem R Gonzales D Auclair F , and others. 2010. The transformation of a unilateral locomotor command into a symmetrical bilateral activation in the brainstem. J Neurosci 30:523–33. 10.1523/JNEUROSCI.3433-09.2010 PMC6632989 20071515

[bibr13-10738584221139136] Buchanan JT Grillner S . 1987. Newly identified “glutamate interneurons” and their role in locomotion in the lamprey spinal cord. Science 236:312–4. 10.1126/science.3563512 3563512

[bibr14-10738584221139136] Cabelguen J-M Bourcier-Lucas C Dubuc R . 2003. Bimodal locomotion elicited by electrical stimulation of the midbrain in the salamander *Notophthalmus viridescens*. J Neurosci 23:2434–9. 10.1523/JNEUROSCI.23-06-02434.2003 PMC6741995 12657703

[bibr15-10738584221139136] Caggiano V Leiras R Goñi-Erro H Masini D Bellardita C Bouvier J , and others. 2018. Midbrain circuits that set locomotor speed and gait selection. Nature 553:455–60. 10.1038/nature25448 PMC5937258 29342142

[bibr16-10738584221139136] Capelli P Pivetta C Soledad Esposito M Arber S . 2017. Locomotor speed control circuits in the caudal brainstem. Nature 551:373–7. 10.1038/nature24064 29059682

[bibr17-10738584221139136] Carbo-Tano M Lapoix M Jia X Auclair F Dubuc R Wyart C . 2022. Functional coupling of the mesencephalic locomotor region and V2a reticulospinal neurons driving forward locomotion. Preprint available from https://www.biorxiv.org/content/10.1101/2022.04.01.486703v1

[bibr18-10738584221139136] Carvalho MM Tanke N Kropff E Witter MP Moser M-B Moser EI . 2020. A brainstem locomotor circuit drives the activity of speed cells in the medial entorhinal cortex. Cell Rep 32:108123. 32905779 10.1016/j.celrep.2020.108123 PMC7487772

[bibr19-10738584221139136] Chambers NE Coyle M Sergio J Lanza K Saito C Topping B , and others. 2021. Effects of pedunculopontine nucleus cholinergic lesion on gait and dyskinesia in hemiparkinsonian rats. Euro J Neurosci 53:2835–47. 10.1111/ejn.15106 33426708

[bibr20-10738584221139136] Chang SJ Cajigas I Guest JD Noga BR Widerström-Noga E Haq I , and others. 2021. MR tractography-based targeting and physiological identification of the cuneiform nucleus for directional DBS in a Parkinson’s disease patient with levodopa-resistant freezing of gait. Front Hum Neurosci 15:676755. 34168545 10.3389/fnhum.2021.676755 PMC8217631

[bibr21-10738584221139136] Chang SJ Cajigas I Opris I Guest JD Noga BR . 2020. Dissecting brainstem locomotor circuits: converging evidence for cuneiform nucleus stimulation. Front Syst Neurosci 14:64. 32973468 10.3389/fnsys.2020.00064 PMC7473103

[bibr22-10738584221139136] Chang SJ Santamaria AJ Sanchez FJ Villamil LM Saraiva PP Benavides F , and others. 2021. Deep brain stimulation of midbrain locomotor circuits in the freely moving pig. Brain Stimul 14:467–76. 10.1016/j.brs.2021.02.017 PMC9097921 33652130

[bibr23-10738584221139136] Chari A Hentall ID Papadopoulos MC Pereira EAC . 2017. Surgical neurostimulation for spinal cord injury. Brain Sci 7:E18. 10.3390/brainsci7020018 PMC5332961 28208601

[bibr24-10738584221139136] Correia PA Lottem E Banerjee D Machado AS Carey MR Mainen ZF . 2017. Transient inhibition and long-term facilitation of locomotion by phasic optogenetic activation of serotonin neurons. Elife 6:e20975. 10.7554/eLife.20975 PMC5308893 28193320

[bibr25-10738584221139136] Cregg JM Leiras R Montalant A Wanken P Wickersham IR Kiehn O . 2020. Brainstem neurons that command mammalian locomotor asymmetries. Nat Neurosci 23:730–40. 10.1038/s41593-020-0633-7 PMC7610510 32393896

[bibr26-10738584221139136] da Silva JA Tecuapetla F Paixão V Costa RM . 2018. Dopamine neuron activity before action initiation gates and invigorates future movements. Nature 554:244–8. 10.1038/nature25457 29420469

[bibr27-10738584221139136] Dautan D Huerta-Ocampo I Gut NK Valencia M Kondabolu K Kim Y , and others. 2020. Cholinergic midbrain afferents modulate striatal circuits and shape encoding of action strategies. Nat Commun 11:1739. 32269213 10.1038/s41467-020-15514-3 PMC7142106

[bibr28-10738584221139136] Dautan D Huerta-Ocampo I Witten IB Deisseroth K Bolam JP Gerdjikov T , and others. 2014. A major external source of cholinergic innervation of the striatum and nucleus accumbens originates in the brainstem. J Neurosci 34:4509–18. 10.1523/JNEUROSCI.5071-13.2014 PMC3965779 24671996

[bibr29-10738584221139136] Dautan D Kovács A Bayasgalan T Diaz-Acevedo MA Pal B Mena-Segovia J . 2021. Modulation of motor behavior by the mesencephalic locomotor region. Cell Rep 36:109594. 34433068 10.1016/j.celrep.2021.109594 PMC8641693

[bibr30-10738584221139136] Dautan D Souza AS Huerta-Ocampo I Valencia M Assous M Witten IB , and others. 2016. Segregated cholinergic transmission modulates dopamine neurons integrated in distinct functional circuits. Nat Neurosci 19:1025–33. 10.1038/nn.4335 PMC5086413 27348215

[bibr31-10738584221139136] Demain A Westby GWM Fernandez-Vidal S Karachi C Bonneville F Do MC , and others. 2014. High-level gait and balance disorders in the elderly: a midbrain disease? J Neurol 261:196–206. 10.1007/s00415-013-7174-x PMC3895186 24202784

[bibr32-10738584221139136] De Silva SR Moore AT . 2022. Optogenetic approaches to therapy for inherited retinal degenerations. J Physiol 600: 4623–32. 10.1113/JP282076 PMC9804934 35908243

[bibr33-10738584221139136] Duty S Jenner P . 2011. Animal models of Parkinson’s disease: a source of novel treatments and clues to the cause of the disease. Br J Pharmacol 164:1357–91. 10.1111/j.1476-5381.2011.01426.x PMC3229766 21486284

[bibr34-10738584221139136] Eidelberg E Walden JG Nguyen LH . 1981. Locomotor control in macaque monkeys. Brain 104:647–63. 10.1093/brain/104.4.647-a 7326562

[bibr35-10738584221139136] Ferreira-Pinto MJ Kanodia H Falasconi A Sigrist M Esposito MS Arber S . 2021. Functional diversity for body actions in the mesencephalic locomotor region. Cell 184:4564–78.e18. 10.1016/j.cell.2021.07.002 PMC8382160 34302739

[bibr36-10738584221139136] Ferreira-Pinto MJ Ruder L Capelli P Arber S . 2018. Connecting circuits for supraspinal control of locomotion. Neuron 100:361–74. 10.1016/j.neuron.2018.09.015 30359602

[bibr37-10738584221139136] Fluri F Malzahn U Homola GA Schuhmann MK Kleinschnitz C Volkmann J . 2017. Stimulation of the mesencephalic locomotor region for gait recovery after stroke. Ann Neurol 82:828–40. 10.1002/ana.25086 29059697

[bibr38-10738584221139136] Fougère M Flaive A Frigon A Ryczko D . 2019. Descending dopaminergic control of brainstem locomotor circuits. Curr Opin Physiol 8:30–5.

[bibr39-10738584221139136] Fougère M van der Zouwen CI Boutin J Neszvecsko K Sarret P Ryczko D . 2021. Optogenetic stimulation of glutamatergic neurons in the cuneiform nucleus controls locomotion in a mouse model of Parkinson’s disease. Proc Natl Acad Sci U S A 118:e2110934118. 10.1073/pnas.2110934118 PMC8639376 34670837

[bibr40-10738584221139136] Galtieri DJ Estep CM Wokosin DL Traynelis S Surmeier DJ . 2017. Pedunculopontine glutamatergic neurons control spike patterning in substantia nigra dopaminergic neurons. Elife 6:e30352. 10.7554/eLife.30352 PMC5643088 28980939

[bibr41-10738584221139136] Garcia-Rill E Houser CR Skinner RD Smith W Woodward DJ . 1987. Locomotion-inducing sites in the vicinity of the pedunculopontine nucleus. Brain Res Bull 18:731–8. 10.1016/0361-9230(87)90208-5 3304544

[bibr42-10738584221139136] Garcia-Rill E Saper CB Rye DB Kofler M Nonnekes J Lozano A , and others. 2019. Focus on the pedunculopontine nucleus: consensus review from the May 2018 Brainstem Society meeting in Washington, DC, USA. Clin Neurophysiol 130:925–40. 10.1016/j.clinph.2019.03.008 PMC7365492 30981899

[bibr43-10738584221139136] Gariépy J-F Missaghi K Chevallier S Chartré S Robert M Auclair F , and others. 2012. Specific neural substrate linking respiration to locomotion. Proc Natl Acad Sci U S A 109:E84–92. 10.1073/pnas.1113002109 PMC3258635 22160700

[bibr44-10738584221139136] Gauvain G Akolkar H Chaffiol A Arcizet F Khoei MA Desrosiers M , and others. 2021. Optogenetic therapy: high spatiotemporal resolution and pattern discrimination compatible with vision restoration in non-human primates. Commun Biol 4:125. 33504896 10.1038/s42003-020-01594-w PMC7840970

[bibr45-10738584221139136] Gay M Belaid H Rogers A Pérez-García F Roustan M Bardinet E , and others. 2020. Anatomo-functional mapping of the primate mesencephalic locomotor region using stereotactic lesions. Mov Disord 35:789–99. 10.1002/mds.27983 31922282

[bibr46-10738584221139136] Goetz L Bhattacharjee M Ferraye MU Fraix V Maineri C Nosko D , and others. 2019. Deep brain stimulation of the pedunculopontine nucleus area in Parkinson disease: MRI-based anatomoclinical correlations and optimal target. Neurosurgery 84:506–18. 10.1093/neuros/nyy151 29846707

[bibr47-10738584221139136] Goetz L Piallat B Bhattacharjee M Mathieu H David O Chabardès S . 2016. On the role of the pedunculopontine nucleus and mesencephalic reticular formation in locomotion in nonhuman primates. J Neurosci 36:4917–29. 10.1523/JNEUROSCI.2514-15.2016 PMC6601854 27147647

[bibr48-10738584221139136] Grätsch S Auclair F Demers O Auguste E Hanna A Büschges A , and others. 2019. A brainstem neural substrate for stopping locomotion. J Neurosci 39:1044–57. 10.1523/JNEUROSCI.1992-18.2018 PMC6363936 30541913

[bibr49-10738584221139136] Grillner S El Manira A . 2020. Current principles of motor control, with special reference to vertebrate locomotion. Physiol Rev 100:271–320. 31512990 10.1152/physrev.00015.2019

[bibr50-10738584221139136] Guillaumin A Serra GP Georges F Wallén-Mackenzie Å . 2021. Experimental investigation into the role of the subthalamic nucleus (STN) in motor control using optogenetics in mice. Brain Res 1755:147226. 33358727 10.1016/j.brainres.2020.147226

[bibr51-10738584221139136] Gut NK Mena-Segovia J . 2019. Dichotomy between motor and cognitive functions of midbrain cholinergic neurons. Neurobiol Dis 128:59–66. 30213733 10.1016/j.nbd.2018.09.008 PMC7176324

[bibr52-10738584221139136] Gut NK Winn P . 2015. Deep brain stimulation of different pedunculopontine targets in a novel rodent model of parkinsonism. J Neurosci 35:4792–803. 10.1523/JNEUROSCI.3646-14.2015 PMC4389588 25810510

[bibr53-10738584221139136] Gut NK Yilmaz D Kondabolu K Huerta-Ocampo I Mena-Segovia J . 2022. Selective inhibition of goal-directed actions in the mesencephalic locomotor region. Preprint available from https://www.biorxiv.org/content/10.1101/2022.01.18.476772v1

[bibr54-10738584221139136] Hérent C Diem S Fortin G Bouvier J . 2021. Upregulation of breathing rate during running exercise by central locomotor circuits. Preprint available from https://www.biorxiv.org/content/10.1101/2021.07.28.453983v1 10.1038/s41467-023-38583-6 PMC10203288 37217517

[bibr55-10738584221139136] Hofer A-S Scheuber MI Sartori AM Good N Stalder SA Hammer N , and others. 2022. Stimulation of the cuneiform nucleus enables training and boosts recovery after spinal cord injury. Brain 145:3681–97. 10.1093/brain/awac184 PMC9586551 35583160

[bibr56-10738584221139136] Howe MW Dombeck DA . 2016. Rapid signalling in distinct dopaminergic axons during locomotion and reward. Nature 535:505–10. 10.1038/nature18942 PMC4970879 27398617

[bibr57-10738584221139136] Huerta-Ocampo I Hacioglu-Bay H Dautan D Mena-Segovia J . 2020. Distribution of midbrain cholinergic axons in the thalamus. eNeuro 7:ENEURO.0454-19.2019. 10.1523/ENEURO.0454-19.2019 PMC6957312 31882534

[bibr58-10738584221139136] Josset N Roussel M Lemieux M Lafrance-Zoubga D Rastqar A Bretzner F . 2018. Distinct contributions of mesencephalic locomotor region nuclei to locomotor control in the freely behaving mouse. Curr Biol 28:884–901.e3. 10.1016/j.cub.2018.02.007 29526593

[bibr59-10738584221139136] Juvin L Grätsch S Trillaud-Doppia E Gariépy J-F Büschges A Dubuc R . 2016. A specific population of reticulospinal neurons controls the termination of locomotion. Cell Rep 15:2377–86. 10.1016/j.celrep.2016.05.029 27264174

[bibr60-10738584221139136] Karachi C Grabli D Bernard FA Tandé D Wattiez N Belaid H , and others. 2010. Cholinergic mesencephalic neurons are involved in gait and postural disorders in Parkinson disease. J Clin Invest 120:2745–54. 10.1172/JCI42642 PMC2912198 20628197

[bibr61-10738584221139136] Kim CK Adhikari A Deisseroth K . 2017. Integration of optogenetics with complementary methodologies in systems neuroscience. Nat Rev Neurosci 18:222–35. 10.1038/nrn.2017.15 PMC5708544 28303019

[bibr62-10738584221139136] Klug JR Engelhardt MD Cadman CN Li H Smith JB Ayala S , and others. 2018. Differential inputs to striatal cholinergic and parvalbumin interneurons imply functional distinctions. eLife 7:e35657. 10.7554/eLife.35657 PMC5929909 29714166

[bibr63-10738584221139136] Korte SM Jaarsma D Luiten PG Bohus B . 1992. Mesencephalic cuneiform nucleus and its ascending and descending projections serve stress-related cardiovascular responses in the rat. J Auton Nerv Syst 41:157–76. 10.1016/0165-1838(92)90137-6 1491112

[bibr64-10738584221139136] Kreeger LJ Connelly CJ Mehta P Zemelman BV Golding NL . 2021. Excitatory cholecystokinin neurons of the midbrain integrate diverse temporal responses and drive auditory thalamic subdomains. Proc Natl Acad Sci U S A 118:e2007724118. 10.1073/pnas.2007724118 PMC7958253 33658359

[bibr65-10738584221139136] Kroeger D Ferrari LL Petit G Mahoney CE Fuller PM Arrigoni E , and others. 2017. Cholinergic, glutamatergic, and GABAergic neurons of the pedunculopontine tegmental nucleus have distinct effects on sleep/wake behavior in mice. J Neurosci 37:1352–66. 10.1523/JNEUROSCI.1405-16.2016 PMC5296799 28039375

[bibr66-10738584221139136] Kuo S-H Kenney C Jankovic J . 2008. Bilateral pedunculopontine nuclei strokes presenting as freezing of gait. Mov Disord 23:616–9. 10.1002/mds.21917 18181207

[bibr67-10738584221139136] Lee AM Hoy JL Bonci A Wilbrecht L Stryker MP Niell CM . 2014. Identification of a brainstem circuit regulating visual cortical state in parallel with locomotion. Neuron 83:455–66. 10.1016/j.neuron.2014.06.031 PMC4151326 25033185

[bibr68-10738584221139136] Leimbach F Gratwicke J Foltynie T Limousin P Zrinzo L Jahanshahi M . 2019. The effects of deep brain stimulation of the pedunculopontine nucleus on cognition in Parkinson’s disease and progressive supranuclear palsy. Clin Park Relat Disord 1:48–51. 34316599 10.1016/j.prdoa.2019.08.001 PMC8288563

[bibr69-10738584221139136] Leiras R Cregg JM Kiehn O . 2022. Brainstem circuits for locomotion. Annu Rev Neurosci 45:63–85. 34985919 10.1146/annurev-neuro-082321-025137

[bibr70-10738584221139136] Lemieux M Bretzner F . 2019. Glutamatergic neurons of the gigantocellular reticular nucleus shape locomotor pattern and rhythm in the freely behaving mouse. PLoS Biol 17:e2003880. 10.1371/journal.pbio.2003880 PMC6502437 31017885

[bibr71-10738584221139136] Le Ray D Brocard F Bourcier-Lucas C Auclair F Lafaille P Dubuc R . 2003. Nicotinic activation of reticulospinal cells involved in the control of swimming in lampreys. Eur J Neurosci 17:137–48. 10.1046/j.1460-9568.2003.02417.x 12534977

[bibr72-10738584221139136] MacLaren DAA Santini JA Russell AL Markovic T Clark SD . 2014. Deficits in motor performance after pedunculopontine lesions in rats—impairment depends on demands of task. Eur J Neurosci 40:3224–36. 10.1111/ejn.12666 24995993

[bibr73-10738584221139136] Mancini M Chung K Zajack A Martini DN Ramsey K Lapidus J , and others. 2019. Effects of augmenting cholinergic neurotransmission on balance in Parkinson’s disease. Parkinsonism Relat Disord 69:40–7. 10.1016/j.parkreldis.2019.10.022 31675664

[bibr74-10738584221139136] Martinez-Gonzalez C Bolam JP Mena-Segovia J . 2011. Topographical organization of the pedunculopontine nucleus. Front Neuroanat 5:22. 21503154 10.3389/fnana.2011.00022 PMC3074429

[bibr75-10738584221139136] Masdeu JC Alampur U Cavaliere R Tavoulareas G . 1994. Astasia and gait failure with damage of the pontomesencephalic locomotor region. Ann Neurol 35:619–21. 10.1002/ana.410350517 8179307

[bibr76-10738584221139136] Masini D Kiehn O . 2022. Targeted activation of midbrain neurons restores locomotor function in mouse models of parkinsonism. Nat Commun 13:504. 35082287 10.1038/s41467-022-28075-4 PMC8791953

[bibr77-10738584221139136] Mazzone P Lozano A Stanzione P Galati S Scarnati E Peppe A , and others. 2005. Implantation of human pedunculopontine nucleus: a safe and clinically relevant target in Parkinson’s disease. Neuroreport 16:1877–81. 10.1097/01.wnr.0000187629.38010.12 16272871

[bibr78-10738584221139136] Mena-Segovia J Bolam JP . 2017. Rethinking the pedunculopontine nucleus: from cellular organization to function. Neuron 94:7–18. 28384477 10.1016/j.neuron.2017.02.027

[bibr79-10738584221139136] Mena-Segovia J Sims HM Magill PJ Bolam JP . 2008. Cholinergic brainstem neurons modulate cortical gamma activity during slow oscillations. J Physiol 586:2947–60. 10.1113/jphysiol.2008.153874 PMC2517196 18440991

[bibr80-10738584221139136] Meng X Wang W Lu H He L-J Chen W Chao ES , and others. 2016. Manipulations of MeCP2 in glutamatergic neurons highlight their contributions to Rett and other neurological disorders. Elife 5:e14199. 10.7554/eLife.14199 PMC4946906 27328325

[bibr81-10738584221139136] Musienko PE Zelenin PV Lyalka VF Orlovsky GN Deliagina TG . 2008. Postural performance in decerebrated rabbit. Behav Brain Res 190:124–34. 10.1016/j.bbr.2008.02.011 PMC2365477 18359100

[bibr82-10738584221139136] Netzer F Sevoz-Couche C . 2021. Rostral cuneiform nucleus and the defence reaction: direct and indirect midbrain-medullary 5-HT mechanisms in baroreflex inhibition. Br J Pharmacol 178:1819–35. 10.1111/bph.15406 33543768

[bibr83-10738584221139136] Ni K-M Hou X-J Yang C-H Dong P Li Y Zhang Y , and others. 2016. Selectively driving cholinergic fibers optically in the thalamic reticular nucleus promotes sleep. Elife 5:e10382. 10.7554/eLife.10382 PMC4764559 26880556

[bibr84-10738584221139136] Noga BR Guest JD . 2021. Combined neuromodulatory approaches in the central nervous system for treatment of spinal cord injury. Curr Opin Neurol 34:804–11. 10.1097/WCO.0000000000000999 PMC8595808 34593718

[bibr85-10738584221139136] Noga BR Whelan PJ . 2022. The mesencephalic locomotor region: beyond locomotor control. Front Neural Circuits 16:884785. 35615623 10.3389/fncir.2022.884785 PMC9124768

[bibr86-10738584221139136] Norton ABW Jo YS Clark EW Taylor CA Mizumori SJY . 2011. Independent neural coding of reward and movement by pedunculopontine tegmental nucleus neurons in freely navigating rats. Eur J Neurosci 33:1885–96. 10.1111/j.1460-9568.2011.07649.x PMC3095748 21395868

[bibr87-10738584221139136] Opris I Dai X Johnson DMG Sanchez FJ Villamil LM Xie S , and others. 2019. Activation of brainstem neurons during mesencephalic locomotor region-evoked locomotion in the cat. Front Syst Neurosci 13:69. 31798423 10.3389/fnsys.2019.00069 PMC6868058

[bibr88-10738584221139136] Ostrem JL Christine CW Glass GA Schrock LE Starr PA . 2010. Pedunculopontine nucleus deep brain stimulation in a patient with primary progressive freezing gait disorder. Stereotact Funct Neurosurg 88:51–5. 10.1159/000268742 20051710

[bibr89-10738584221139136] Pernia-Andrade AJ Wenger N Esposito MS Tovote P . 2021. Circuits for state-dependent modulation of locomotion. Front Hum Neurosci 15:745689. 34858153 10.3389/fnhum.2021.745689 PMC8631332

[bibr90-10738584221139136] Petzold A Valencia M Pál B Mena-Segovia J . 2015. Decoding brain state transitions in the pedunculopontine nucleus: cooperative phasic and tonic mechanisms. Front Neural Circuits 9:68. 26582977 10.3389/fncir.2015.00068 PMC4628121

[bibr91-10738584221139136] Pienaar IS Gartside SE Sharma P De Paola V Gretenkord S Withers D , and others. 2015. Pharmacogenetic stimulation of cholinergic pedunculopontine neurons reverses motor deficits in a rat model of Parkinson’s disease. Mol Neurodegener 10:47. 26394842 10.1186/s13024-015-0044-5 PMC4580350

[bibr92-10738584221139136] Pienaar IS Harrison IF Elson JL Bury A Woll P Simon AK , and others. 2015. An animal model mimicking pedunculopontine nucleus cholinergic degeneration in Parkinson’s disease. Brain Struct Funct 220:479–500. 24292256 10.1007/s00429-013-0669-5

[bibr93-10738584221139136] Plaha P Gill SS . 2005. Bilateral deep brain stimulation of the pedunculopontine nucleus for Parkinson’s disease. Neuroreport 16:1883–7. 10.1097/01.wnr.0000187637.20771.a0 16272872

[bibr94-10738584221139136] Ratner M . 2021. Light-activated genetic therapy to treat blindness enters clinic. Nat Biotechnol 39:126–7. 10.1038/s41587-021-00823-9 33564161

[bibr95-10738584221139136] Richardson M . 2014. Deep brain stimulation for locomotor recovery following spinal cord injury. Neurosurgery 74: N18–9. 10.1227/01.neu.0000442979.07078.ac 24435148

[bibr96-10738584221139136] Roseberry TK Lee AM Lalive AL Wilbrecht L Bonci A Kreitzer AC . 2016. Cell-type-specific control of brainstem locomotor circuits by basal ganglia. Cell 164:526–37. 10.1016/j.cell.2015.12.037 PMC4733247 26824660

[bibr97-10738584221139136] Roussel M Lafrance-Zoubga D Josset N Lemieux M Bretzner F . 2022. Functional contribution of mesencephalic locomotor region nuclei to locomotor recovery after spinal cord injury. Preprint available from https://www.biorxiv.org/content/10.1101/2022.08.22.504420v1 10.1016/j.xcrm.2023.100946 PMC9975330 36812893

[bibr98-10738584221139136] Roth BL . 2016. DREADDs for neuroscientists. Neuron 89:683–94. 10.1016/j.neuron.2016.01.040 PMC4759656 26889809

[bibr99-10738584221139136] Ruan Y Li K-Y Zheng R Yan Y-Q Wang Z-X Chen Y , and others. 2022. Cholinergic neurons in the pedunculopontine nucleus guide reversal learning by signaling the changing reward contingency. Cell Rep 38:110437. 35235804 10.1016/j.celrep.2022.110437

[bibr100-10738584221139136] Ryczko D Auclair F Cabelguen J-M Dubuc R . 2016. The mesencephalic locomotor region sends a bilateral glutamatergic drive to hindbrain reticulospinal neurons in a tetrapod. J Comp Neurol 524:1361–83. 10.1002/cne.23911 PMC5019149 26470600

[bibr101-10738584221139136] Ryczko D Cone JJ Alpert MH Goetz L Auclair F Dubé C , and others. 2016. A descending dopamine pathway conserved from basal vertebrates to mammals. Proc Natl Acad Sci U S A 113:E2440–9. 10.1073/pnas.1600684113 PMC4855556 27071118

[bibr102-10738584221139136] Ryczko D Dubuc R . 2013. The multifunctional mesencephalic locomotor region. Curr Pharm Des 19:4448–70. 10.2174/1381612811319240011 23360276

[bibr103-10738584221139136] Ryczko D Dubuc R . 2017. Dopamine and the brainstem locomotor networks: from lamprey to human. Front Neurosci 11:295. 28603482 10.3389/fnins.2017.00295 PMC5445171

[bibr104-10738584221139136] Ryczko D Grätsch S Alpert MH Cone JJ Kasemir J Ruthe A , and others. 2020. Descending dopaminergic inputs to reticulospinal neurons promote locomotor movements. J Neurosci 40:8478–90. 10.1523/JNEUROSCI.2426-19.2020 PMC7605428 32998974

[bibr105-10738584221139136] Sahel J-A Boulanger-Scemama E Pagot C Arleo A Galluppi F Martel JN , and others. 2021. Partial recovery of visual function in a blind patient after optogenetic therapy. Nat Med 27:1223–9. 10.1038/s41591-021-01351-4 34031601

[bibr106-10738584221139136] Scelzo E Lozano AM Hamani C Poon Y-Y Aldakheel A Zadikoff C , and others. 2017. Peduncolopontine nucleus stimulation in progressive supranuclear palsy: a randomised trial. J Neurol Neurosurg Psychiatry 88:613–6. 10.1136/jnnp-2016-315192 28214797

[bibr107-10738584221139136] Schweizer N Pupe S Arvidsson E Nordenankar K Smith-Anttila CJA Mahmoudi S , and others. 2014. Limiting glutamate transmission in a Vglut2-expressing subpopulation of the subthalamic nucleus is sufficient to cause hyperlocomotion. Proc Natl Acad Sci U S A 111:7837–42. 10.1073/pnas.1323499111 PMC4040590 24821804

[bibr108-10738584221139136] Sébille SB Rolland A-S Faillot M Perez-Garcia F Colomb-Clerc A Lau B , and others. 2019. Normal and pathological neuronal distribution of the human mesencephalic locomotor region. Mov Disord 34:218–27. 10.1002/mds.27578 30485555

[bibr109-10738584221139136] Sharma PK Wells L Rizzo G Elson JL Passchier J Rabiner EA , and others. 2020. DREADD activation of pedunculopontine cholinergic neurons reverses motor deficits and restores striatal dopamine signaling in parkinsonian rats. Neurotherapeutics 17:1120–41. 10.1007/s13311-019-00830-4 PMC7609798 31965550

[bibr110-10738584221139136] Shik ML Severin FV Orlovskiĭ GN . 1966. Control of walking and running by means of electric stimulation of the midbrain. Article in Russian. Biofizika 11:659–66. 6000625

[bibr111-10738584221139136] Shine JM Matar E Ward PB Bolitho SJ Gilat M Pearson M , and others. 2013. Exploring the cortical and subcortical functional magnetic resonance imaging changes associated with freezing in Parkinson’s disease. Brain 136:1204–15. 10.1093/brain/awt049 23485851

[bibr112-10738584221139136] Sirota MG Di Prisco GV Dubuc R . 2000. Stimulation of the mesencephalic locomotor region elicits controlled swimming in semi-intact lampreys. Eur J Neurosci 12: 4081–92. 10.1046/j.1460-9568.2000.00301.x 11069605

[bibr113-10738584221139136] Smetana R Juvin L Dubuc R Alford S . 2010. A parallel cholinergic brainstem pathway for enhancing locomotor drive. Nat Neurosci 13:731–8. 10.1038/nn.2548 PMC2881475 20473293

[bibr114-10738584221139136] Steeves JD Sholomenko GN Webster DM . 1987. Stimulation of the pontomedullary reticular formation initiates locomotion in decerebrate birds. Brain Res 401:205–12. 10.1016/0006-8993(87)91406-5 3815097

[bibr115-10738584221139136] Stieglitz LH Hofer A-S Bolliger M Oertel MF Filli L Willi R , and others. 2021. Deep brain stimulation for locomotion in incomplete human spinal cord injury (DBS-SCI): protocol of a prospective one-armed multi-centre study. BMJ Open 11:e047670. 10.1136/bmjopen-2020-047670 PMC8487195 34593490

[bibr116-10738584221139136] Thevathasan W Debu B Aziz T Bloem BR Blahak C Butson C , and others;, and Movement Disorders Society PPN DBS Working Group in collaboration with the World Society for Stereotactic and Functional Neurosurgery. 2018. Pedunculopontine nucleus deep brain stimulation in Parkinson’s disease: a clinical review. Mov Disord 33:10–20. 28960543 10.1002/mds.27098

[bibr117-10738584221139136] Thevathasan W Pogosyan A Hyam JA Jenkinson N Foltynie T Limousin P , and others. 2012. Alpha oscillations in the pedunculopontine nucleus correlate with gait performance in parkinsonism. Brain 135:148–60. 10.1093/brain/awr315 PMC3267984 22232591

[bibr118-10738584221139136] Usseglio G Gatier E Heuzé A Hérent C Bouvier J . 2020. Control of orienting movements and locomotion by projection-defined subsets of brainstem V2a neurons. Curr Biol 30:4665–81.e6. 10.1016/j.cub.2020.09.014 33007251

[bibr119-10738584221139136] van der Zouwen CI Boutin J Fougère M Flaive A Vivancos M Santuz A , and others. 2021. Freely behaving mice can brake and turn during optogenetic stimulation of the mesencephalic locomotor region. Front Neural Circuits 15:639900. 33897379 10.3389/fncir.2021.639900 PMC8062873

[bibr120-10738584221139136] Xiao C Cho JR Zhou C Treweek JB Chan K McKinney SL , and others. 2016. Cholinergic mesopontine signals govern locomotion and reward through dissociable midbrain pathways. Neuron 90:333–47. 10.1016/j.neuron.2016.03.028 PMC4840478 27100197

[bibr121-10738584221139136] Xiao H Li M Cai J Li N Zhou M Wen P , and others. 2017. Selective cholinergic depletion of pedunculopontine tegmental nucleus aggravates freezing of gait in parkinsonian rats. Neurosci Lett 659:92–8. 10.1016/j.neulet.2017.08.016 28803956

[bibr122-10738584221139136] Wang X Zhang C Szábo G Sun Q-Q . 2013. Distribution of CaMKIIα expression in the brain in vivo, studied by CaMKIIα-GFP mice. Brain Res 1518:9–25. 23632380 10.1016/j.brainres.2013.04.042 PMC3747672

[bibr123-10738584221139136] Wilcox RA Cole MH Wong D Coyne T Silburn P Kerr G . 2011. Pedunculopontine nucleus deep brain stimulation produces sustained improvement in primary progressive freezing of gait. J Neurol Neurosurg Psychiatry 82:1256–9. 10.1136/jnnp.2010.213462 20971757

[bibr124-10738584221139136] Yoo JH Zell V Wu J Punta C Ramajayam N Shen X , and others. 2017. Activation of pedunculopontine glutamate neurons is reinforcing. J Neurosci 37:38–46. 28053028 10.1523/JNEUROSCI.3082-16.2016 PMC5214635

